# A Generic Model to Estimate Wheat LAI over Growing Season Regardless of the Soil-Type Background

**DOI:** 10.34133/plantphenomics.0055

**Published:** 2023-05-23

**Authors:** Qiaomin Chen, Bangyou Zheng, Karine Chenu, Scott C. Chapman

**Affiliations:** ^1^ School of Agriculture and Food Sciences, The University of Queensland, St Lucia, QLD, Australia.; ^2^ Agriculture and Food, CSIRO, Queensland Bioscience Precinct, St Lucia, QLD, Australia.; ^3^ The University of Queensland, Queensland Alliance for Agriculture and Food Innovation, Toowoomba, QLD, Australia.

## Abstract

It is valuable to develop a generic model that can accurately estimate the leaf area index (LAI) of wheat from unmanned aerial vehicle-based multispectral data for diverse soil backgrounds without any ground calibration. To achieve this objective, 2 strategies were investigated to improve our existing random forest regression (RFR) model, which was trained with simulations from a radiative transfer model (PROSAIL). The 2 strategies consisted of (a) broadening the reflectance domain of soil background to generate training data and (b) finding an appropriate set of indicators (band reflectance and/or vegetation indices) as inputs of the RFR model. The RFR models were tested in diverse soils representing varying soil types in Australia. Simulation analysis indicated that adopting both strategies resulted in a generic model that can provide accurate estimation for wheat LAI and is resistant to changes in soil background. From validation on 2 years of field trials, this model achieved high prediction accuracy for LAI over the entire crop cycle (LAI up to 7 m^2^ m^−2^) (root mean square error (RMSE): 0.23 to 0.89 m^2^ m^−2^), including for sparse canopy (LAI less than 0.3 m^2^ m^−2^) grown on different soil types (RMSE: 0.02 to 0.25 m^2^ m^−2^). The model reliably captured the seasonal pattern of LAI dynamics for different treatments in terms of genotypes, plant densities, and water–nitrogen managements (correlation coefficient: 0.82 to 0.98). With appropriate adaptations, this framework can be adjusted to any type of sensors to estimate various traits for various species (including but not limited to LAI of wheat) in associated disciplines, e.g., crop breeding, precision agriculture, etc.

## Introduction

The estimation of leaf area index (LAI) has long been intensively studied in plant sciences, agronomy, and remote sensing communities. The LAI sets the canopy capacity for crop photosynthesis and transpiration and can be used as an indicator of crop health condition and crop growth rate. The direct measurement of LAI is destructive, labor-intensive, and time-consuming but more accurate; thus, it is commonly used to provide ground truth for developing indirect methods [1,2]. With the rapid development in technologies related to imaging and geopositioning, as well as data management, processing, and analysis, many indirect methods have been proposed and validated to retrieve LAI from sensing data (e.g., RGB, multispectral, and hyperspectral) captured from various phenotyping platforms including satellites, drones, ground-based stations, or vehicles [3–5]. In most studies, LAI refers to green LAI, but when retrieved from spectral images, it may represent the green area index (GAI), i.e., corresponding to all green parts of plants including leaves, stems, and heads [6,7].

Retrieval methods to predict crop traits (e.g., LAI) from sensing data are independent from cameras (or sensors) and platforms used to capture sensing data. According to the means to establish the relationship between spectral signal and crop trait, these methods can be divided into 3 main categories: (a) empirical methods, when a relationship between the crop trait and the raw sensing data or/and their derived vegetation indices (VIs) is established with experimental data; (b) physical methods, when this relationship corresponds to the cause–effect relationship built within a radiative transfer model (RTM) (for LAI estimation in particular, including Beer–Lambert’s Law based on gap fraction theory that is the theoretical basic of the indirect estimation of LAI [5]); and (c) hybrid methods, when this relationship is built from RTM simulations [8,9]. The hybrid method is a 2-step method that is to generate a synthetic dataset by running the RTM in a forward mode and then using this dataset to train a predictive model to predict crop traits from sensing data [1,10,11]. There is an increasing interest in developing hybrid methods as they can balance general applicability and computational efficiency. The general applicability of the predictive model in the hybrid methods results from the synthetic dataset (including diverse canopy structures and observation conditions) used for training [12,13]. As reviewed in the literature, different algorithms used to establish the predictive model in hybrid methods have advantages and limitations with no obvious global solution, but machine learning algorithms are assumed to utilize sensing data more sufficiently and flexibly than methods do based on look-up table or numeric optimization [14–17].

The soil background has a substantial effect on plot reflectance for sparse canopies with LAI less than 2 m^2^ of leaf per m^−2^ of land area [18]. In the hybrid method, the soil reflectance used to generate synthetic training data is commonly customized with local soil reflectance to improve the prediction accuracy in local environments [19,20]. In practice, this means that, first, the soil background of each environment needs to be characterized and, second, a soil-specific model needs to be trained to achieve accurate prediction for low LAI. This impedes usage of the method at large scales or across large numbers of sites, even if the local soil reflectance can be easily calibrated from reflectance of soil pixels in images, e.g., using the calibration method proposed by Chen et al. [8]. In addition, soil-specific models are typically less stable when predicting LAI across growing season [20,21] as the background changes over time, e.g., with rainfall (or irrigation) changing the spectral properties of the surface and senescent (non-green) vegetation and plant residues masking the soil toward the end of the season. Soil-specific models are also likely to be less accurate in large fields with high soil-type spatial variability.

In synthetic data, the soil reflectance is also assumed to be associated with a pure soil background [22,23], while the actual background pixel might be a mixture of soil, plant residues, and weeds. In practice, this results in differences between simulation and observation and reduces the prediction accuracy. This problem can be addressed with an image background correction to replace the actual mixed soil background with the pure soil background and keep the vegetation fraction consistent [8]. However, the success of this background correction method relies on the image spatial resolution (or pixel size) and the accuracy of the binary classification of vegetation versus background. Degradation of image spatial resolution theoretically will increase the mixed pixels and decrease the pure vegetation and soil background pixels, hindering the accurate classification of vegetation and background [24]. In practice, whether degradation of image spatial resolution increases the number of mixed pixels depends on the homogeneity within the original image and new resolution after degradation. In particular, degradation in a homogeneous region will not generate mixed pixels. From this aspect, it is challenging to accurately estimate low LAI from drone-based data at centimeter resolution and nearly impossible from satellite-based data at meter resolution.

Although the “background-resistant model” concept has not been explicitly proposed, previous studies have attempted to improve LAI prediction by (a) considering multiple soil reflectance when generating synthetic training data [10,25] and (b) developing better VIs resistant to chlorophyll and background changes [1,26]. In theory, a background-resistant model should effectively address problems mentioned above to achieve stable and accurate LAI prediction over growing season under different soil backgrounds. Furthermore, such a background-resistant model should estimate LAI even from low spatial resolution images, in which canopy reflectance of pixels are equivalent to reflectance comprising a mixture of soil background and vegetation. Such a background-resistant model is particularly useful for LAI estimation in dryland regions such as in Australia, where the crop LAI may rarely exceed 5 m^2^ m^−2^, and the wheat canopy has LAI less than 2 m^2^ m^−2^ for 1 to 3 months after sowing. In sandy soils, the LAI may rarely exceed 2 m^2^ m^−2^ during the whole growing season under low-rainfall rainfed conditions, e.g., in Western Australia.

The stable estimation of LAI retrieved with sensing technologies is strongly affected by the spatiotemporal variations in background caused by spatial variability of soil, seasonal senescence of vegetation, and mixed pixel issues of images. To improve previous work focusing on soil-specific model for predicting wheat LAI at pre-anthesis vegetative stages [8], this research aims to develop a generic machine learning-based prediction model that supports accurate estimation of LAI across diverse soil backgrounds for the entire growth season in field conditions. Research objectives include the following: (a) developing different strategies (i.e., soil reflectance domain extension and canopy-spectral indicator selection) to improve generalization of the prediction model, (b) evaluating performance of the established prediction model under diverse conditions (including different soil backgrounds, LAI levels, and growing stages) using both simulation and experimental data, and (c) accounting for the varying model performance in different conditions.

## Materials and Methods

### Overview

**Fig. 1.**
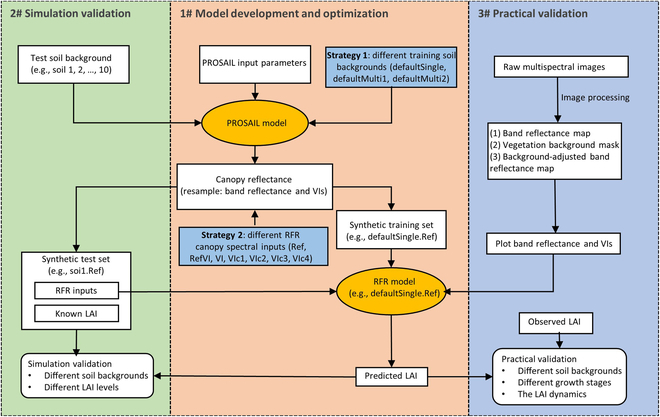
Research flow map. PROSAIL is a radiative transfer model, coupling a leaf optical property model (PROSPECT-D) and a canopy bidirectional reflectance model (4SAIL). The reflectance of the test soil background and the reflectance boundaries of the training soil background are presented in Fig. 3. Both synthetic training sets and synthetic test sets are generated from PROSAIL with the same parameter ranges. The synthetic training sets were used to develop random forest regression (RFR) models, while the synthetic test sets were used to evaluate the simulation performance of RFR models. The practical performance of RFR models was evaluated on experimental data collected from field experiments.

The research flow map of this study is presented in Fig. 1. This study proposes a background-resistant predictive model to predict wheat LAI through investigating the setting of (a) soil reflectance used in synthetic training data (strategy 1) and/or (b) canopy-spectral inputs of random forest regression (RFR) model (strategy 2). In total, 21 RFR models (3 sets of training soil backgrounds × 7 sets of RFR canopy-spectral inputs) were developed. These models were first tested on synthetic test data to evaluate the model’s simulation performance of LAI prediction under different test soil backgrounds (representing the diversity of soil surface reflectance in Australia) for varying LAI levels. Subsequently, these models were tested on experimental and augmented unmanned aerial vehicle (UAV)-based multispectral data representing different soil backgrounds to evaluate model’s practical performance of predicting low LAI at early stage. Finally, these models were also tested on experimental data to evaluate model’s practical performance of predicting LAI at different growing stages and the dynamics of LAI during the whole growing season.

Compared to our previous work that can produce reliable LAI estimation for wheat under a specific soil background based on local soil calibration and image background correction [8], the study provided an improved solution to develop a generic predictive model that can be applied in varying soil backgrounds. The workflow of generating synthetic datasets and training RFR models was adapted from our previous work [8], as were the field experiments for validation. Method details related to these parts (in the “Field experiments,” “Simulating synthetic datasets with PROSAIL model,” and “Developing a baseline model” sections) are more thoroughly explained in the previous paper [8].

### Field experiments

**Fig. 2.**
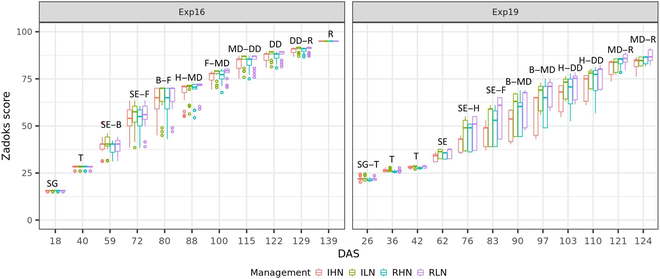
Observed phenology (characterized by Zadoks score) for each plot on each UAV-based phenotyping dates expressed as days after sowing (DAS). The experiments Exp16 and Exp19 were conducted in 2016 and 2019, respectively. The texts above the symbols denote the corresponding growth stage: seedling growth (SG), tillering (T), stem elongation (SE), booting (B), heading (H), flowering (F), milk development (MD), dough development (DD), and ripening (R).

Two wheat experiments were conducted at Gatton, Queensland (27.55°S, 152.33°E) in 2016 (Exp16) and 2019 (Exp19), and UAV-based phenotyping was undertaken along with field measurements (for more details, see Chen et al. [8]). Different genotypes, irrigation, and fertilization regimes were applied to create contrasting canopy structures. In summary, the whole field was split into 4 treatment blocks based on irrigation and fertilization regimes, i.e., irrigation and high nitrogen (IHN), irrigation and low nitrogen (ILN), rainfed and high nitrogen (RHN), as well as rainfed and low nitrogen (RLN). Each block was split into small plots of ~14 m^2^ (2 × 7 m), each with 7 rows and a 25-cm row spacing. For Exp16, the sowing occurred on 2016 May 21 and plant emergence occurred approximately 10 d after sowing. For Exp19, the sowing occurred on 2019 May 15 and plant emergence occurred approximately 5 d after sowing. The phenology of each plot on each UAV-based phenotyping date was recorded using a decimal Zadoks score (Fig. 2) [27].

LAI was measured from quadrat harvests that comprised 0.5 m of length of 4 inner rows (~0.5 m^2^) and were taken in 84 plots (including 7 genotypes, 1 sowing densities, 4 water–nitrogen treatments, and 3 replicates) for Exp16 and 72 plots (including 3 genotypes, 3 sowing densities, 4 water–nitrogen treatments, and 2 replicates) for Exp19. Within each block (defined by water and nitrogen application), all treatments (genotype or genotype × density) were randomized using the corDiGGer method provided in a free R package, DiGGer. For Exp16, quadrat harvest and UAV phenotyping did not always occur within 3 d of each other. Unmatching LAI measurements on UAV phenotyping dates were interpolated with a fitted piecewise function based on all observed LAI from quadrat harvests across growing season (7 or 8 harvesting times were used for each selected plots from leave development to end of grain filling). The piecewise function is consisting of using a logistic function and beta function before and after LAI reaching its maxima, respectively. A detailed description for the implementation of this method can refer to the Supplementary Materials in our previous work [8]. For Exp19, all 4 quadrat harvests (days after sowing (DAS) = 36, 64, 90, and 111) were taken within 2 d after the corresponding UAV phenotyping. Hence, each ground-estimated LAI for each selected plot had one UAV-based estimation. It should be noticed that the UAV-based estimation can represent LAI (only leaves presented in images) or GAI (all green parts including stems and heads except for leaves presented in images), and these terms are used variously in published papers.

All UAV flights were undertaken to capture multispectral data from 10:00 AM to 2:00 PM in clear days without strong wind effects in both Exp16 (DAS = 18, 40, 59, 72, 80, 88, 100, 115, 122, 129, and 139) and Exp19 (DAS = 26, 36, 42, 62, 76, 83, 90, 97, 103, 110, 121, and 124). The flight height was set to 20 m (with a ground sampling distance (GSD) of 1.3 cm) for the first 4 flight in Exp16, and in the first 3 flights in Exp19, 40 m (with a GSD of 2.7 cm) only for the fourth flight in Exp19, as well as 30 m (with a GSD of 2 cm) for the remaining flights in both experiments. The multispectral camera used in this study was a MicaSense RedEdge camera (https://www.micasense.com), with 5 bands in the visible near-infrared (VNIR) range, i.e., blue (475 nm of center wavelength, 20 nm of bandwidth), green (560 nm, 20 nm), red (668 nm, 10 nm), NIR (840 nm, 40 nm), and red edge (717 nm, 10 nm). Raw multispectral images were processed in Pix4Dmapper software (version 4.3.4) (https://www.pix4d.com) to generate the calibrated reflectance map of each band for the whole field, using images of the calibrated reference panel for calibration. According to the field experimental design, each reflectance map was segmented into individual plots. Marginal areas from adjacent plots and plot gaps were trimmed from individual plots by a percentage of 10% along 4 sides. The Exp16 followed a double-plot design (one was used for destructive harvest and an adjacent plot for UAV-based phenotyping), so the UAV-based estimated LAI was retrieved from the phenotyping plot (with an area of approximately 8.96 m^2^). However, the same plot was used for both destructive harvest and UAV-based phenotyping for Exp19. To predict LAI from the same area for each flight in Exp19, the harvested areas were clipped from individual plot images, and the remaining area (approximately 2.24 m^2^) was used to retrieve UAV-based estimated LAI. After processing, each plot image included about 53,444 or 22,400 pixels in Exp16 and 13,392, 5,600, or 3,120 pixels for Exp19, depending on GSD at specific flight height. Finally, the pixel-scale reflectance from the trimmed plot was averaged to generate the plot-scale reflectance and derived plot-scale VIs that were used in predictive models to predict LAI. In this case, the predicted value corresponded to GAI as the green parts contributed from stems and heads were not separated from those of leaves. At the early stage before appearance of heads, the LAI was close to GAI because of the marginal view of stems from the nadir view. For the remainder of this paper, the value retrieved with the predictive model was named as predicted LAI, although in the case of wheat in later stages of growth, this value might be more accurately termed as GAI.

### Simulating synthetic datasets with the PROSAIL model

The PROSAIL model [23] was used to simulate canopy reflectance for given input parameters and soil reflectance. The current version, PROSAIL-D (coupling PROSPECT-D with 4SAIL), used in this study is available online (http://teledetection.ipgp.jussieu.fr/prosail/). To represent possible canopy structures of wheat and observation conditions, 13 input parameters were defined on the basis of our previous work [8]. The parameters and ranges (obey a uniform distribution) were the following: leaf mesophyll structure parameter (Ns; unitless; range [1, 2.5]), leaf chlorophyll content (Cab; μg cm^−2^; [0, 90]), leaf carotenoid content (Car; μg cm^−2^; [0, 20]), leaf water content or leaf equivalent water thickness (Cw; g cm^−2^; [0.001, 0.03]), leaf dry matter content (Cm; g cm^−2^; [0.001, 0.01]), average leaf inclination angle (ALA; degree; [20, 70]), leaf area index (LAI; m^2^ m^-2^; [0, 7]), hot spot parameter (hspot; m m^−1^; [0.01, 0.5]), solar zenith angle (SZA; degree; [20, 70]), and relative azimuth angle (RAA; degree; [−90, 90]). Parameters for the leaf anthocyanins content (Cant; μg cm^−2^), leaf brown pigment (Cbrown; unitless), and viewing zenith angle (VZA; degree) were fixed to 0.

A subset consisting of 40,000 combinations (training: 30,000 samples; test: 10,000 samples) of input parameter values was randomly sampled from the defined parameter space and then further combined with soil reflectance from defined soil backgrounds to run PROSAIL and simulate canopy reflectance. The soil reflectance of the soil background used in the training set was simulated with the default soil reflectance provided in PROSAIL by adjusting 2 soil factors (i.e., psoil and asoil), while that used in the test set was corresponding to a specific measured soil reflectance of test soils (see details in the “Improving the baseline model with “strategy 1”” section). The soil reflectance and the combination of parameter values were combined randomly in each training set. To mimic measurements from the sensor mounted on the UAV platform, the output bidirectional reflectance from 400 to 2,500 nm at 1-nm interval was resampled into band reflectance of the 5 bands based on spectral response coefficient provided by MicaSense (for more details, refer to Chen et al. [8]). The related band reflectance was then used to calculate related VIs (see details in the “Improving the baseline model with “strategy 2”” section). The band reflectance of 5 bands or/and derived VIs were coupled with known LAI (i.e., input used for PROSAIL) to generate paired data and define the synthetic dataset. The synthetic training data (representing a broad range of canopy structures and observation conditions in a specific soil background consisting of single or multiple soil reflectance) were used to train the predictive model that was then tested in diverse situations including multiple “new” soil backgrounds (see details in the “Validation on synthetic data generated with new test soils” section).

### Developing and improving RFR models

#### Developing a baseline model

In this study, the RFR was chosen as the predictive model to predict LAI, as RFR is less prone to overfitting than some non-ensemble methods and can provide robust predictions because of its attributes, i.e., ensemble mechanism, sample disturbance, and attribute disturbance [28]. Three key hyperparameters were identified in the literature [29,30]: (a) the number of trees or base learners (n_estimators = 200), (b) the number of features to consider finding out the best split (max_features = log_2_(n_features), where n_features represents the number of predictive variables of the RFR model), and (c) the minimum number of samples required at a leaf node (min_samples_leaf = 1). The values of these 3 hyperparameter were determined on the basis of grid search and cross-validation as described in our previous work [8]. Both input (i.e., band reflectance or/and VIs) and output variables (i.e., LAI) of the RFR model were normalized with the mean–standard deviation normalization (also called standardization) approach to prevent any scaling issues. The MSE was used to evaluate the model performance during training. The RFR model was implemented with Python 3.7.2 using the scikit-learn open-source machine learning library (version 0.24.2; https://scikit-learn.org/stable/).

The baseline RFR model was trained on synthetic data using the soil background with a single soil reflectance (“defaultSingle”) and using the 5 band reflectance as model inputs (“Ref”) (see details in the “Improving the baseline model with “strategy 1”” and “Improving the baseline model with “strategy 2”” sections for definitions of “defaultSingle” and “Ref,” respectively).

#### Improving the baseline model with “strategy 1”

The strategy 1 (i.e., broadening the reflectance domain of the training soil background) was first investigated to improve the baseline model to perform robustly in different soil backgrounds. PROSAIL provides a default soil with standard soil reflectance under wet (Rsoil_wet_) and dry (Rsoil_dry_) conditions. In PROSAIL, possible variation of soil reflectance can be accounted for by a wetness factor (psoil) (used to mix the wet and dry soil) and a multiplicative brightness factor (asoil) [31]. The reflectance of a particular soil (Rsoil) with specific wetness and brightness relative to the default soil provided in PROSAIL can be calculated from the following equation: Rsoil = asoil × (psoil × Rsoil_dry_ + (1 − psoil) × Rsoil_wet_) [8].

**Fig. 3.**
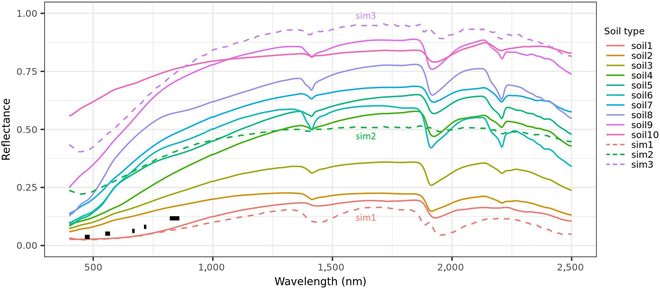
Soil reflectance profiles of the selected soil samples and possible boundaries of the training soil reflectance. The solid lines indicate the soil reflectance used in test tests, while the dashed lines indicate the boundaries of soil reflectance used in training sets. The black segments indicate the actual soil reflectance of 5 bands derived from UAV multispectral images in Exp16.

For strategy 1, 3 sets of soil backgrounds were used in the synthetic training set (i.e., “defaultSingle,” “defaultMulti1,” and “defaultMulti2”). The 3 boundaries of soil reflectance in training soil backgrounds were generated with the equation mentioned above by specifying psoil and asoil: sim1 (psoil = 0, asoil = 1), sim2 (psoil = 1, asoil = 1), and sim3 (psoil = 0.9, asoil = 2) (Fig. 3). The “defaultSingle” soil background corresponded to a single soil reflectance profile equivalent to “sim2.” The “defaultMulti1” soil background corresponded to multiple soil reflectance profiles within the domain between “sim1” (lower boundary) and “sim2” (upper boundary). These soil reflectance profiles were generated by fixing asoil to 1 and changing psoil from 0 to 1. The “defaultMulti2” soil background corresponded to multiple soil reflectance profiles within the domain between “sim1” (lower boundary) and “sim2” (upper boundary). These soil reflectance profiles were generated by changing both psoil (between 0 and 1) and asoil (between 0.5 and 2). The soil reflectance profiles included in each soil background were randomly combined with the combination of parameter values to generate the corresponding training set.

#### Improving the baseline model with “strategy 2”

The strategy 2 (i.e., improving canopy-spectral indicators used as model inputs) was then investigated to improve the baseline model to make it insensitive to changes in soil backgrounds. In this study, in addition to the 5 band reflectance, 7 VIs (Table 1) were considered as canopy-spectral inputs for LAI prediction from multispectral data. For example, the normalized difference vegetation index (NDVI) was selected as it has been intensively used to retrieve LAI despite that it easily saturates for high LAI. The other 6 VIs (i.e., red edge chlorophyll index (CIre), red edge modified simple ratio (MSRre), red edge normalized difference vegetation index (NDVIre), enhanced vegetation index 2 (EVI2), modified chlorophyll absorption ratio index 2 (MCARI2), and modified triangular vegetation index 2 (MTVI2)) were selected as they have been reported or proven to be well correlated to LAI and less sensitive to variations in background, pigment, and canopy structure in the literature [1,26].

**Table 1.** Vegetation indices used in this study. The NIR, R, RE, and G represent the top-of-canopy reflectance in near-infrared, red, red edge, and green bands of the MicaSense RedEdge camera, respectively.

Vegetation index	Equation	Reference
Normalized difference vegetation index (NDVI)	NDVI = (NIR − R)/(NIR + R)	[32]
Red edge chlorophyll index (CIre)	CI_re_ = NIR/RE − 1	[33]
Red edge modified simple ratio (MSRre)	${\mathrm{MSR}}_{\mathrm{re}}=\frac{\mathrm{NIR}/\mathrm{RE}-1}{\sqrt{\mathrm{NIR}/\mathrm{RE}+1}}$	[34]
Red edge normalized difference vegetation index (NDVIre)	NDVI_re_ = (NIR − RE)/(NIR + RE)	[35]
Enhanced vegetation index 2 (EVI2)	EVI2 = (2.5(NIR − R))/(NIR + 2.4R + 1)	[36]
Modified chlorophyll absorption ratio index 2 (MCARI2)	$\mathrm{MCARI}2=\frac{1.5\left(2.5\left(\mathrm{NIR}-R\right)-1.3\left(\mathrm{NIR}-G\right)\right)}{\sqrt{{\left(2\mathrm{NIR}+1\right)}^{2}-\left(6\mathrm{NIR}-5\sqrt{R}\right)-0.5}}$	[1]
Modified triangular vegetation index 2 (MTVI2)	$\mathrm{MTVI}2=\frac{1.5\left(1.2\left(\mathrm{NIR}-G\right)-2.5\left(R-G\right)\right)}{\sqrt{{\left(2\mathrm{NIR}+1\right)}^{2}-\left(6\mathrm{NIR}-5\sqrt{R}\right)-0.5}}$	[1]

**Fig. 4.**
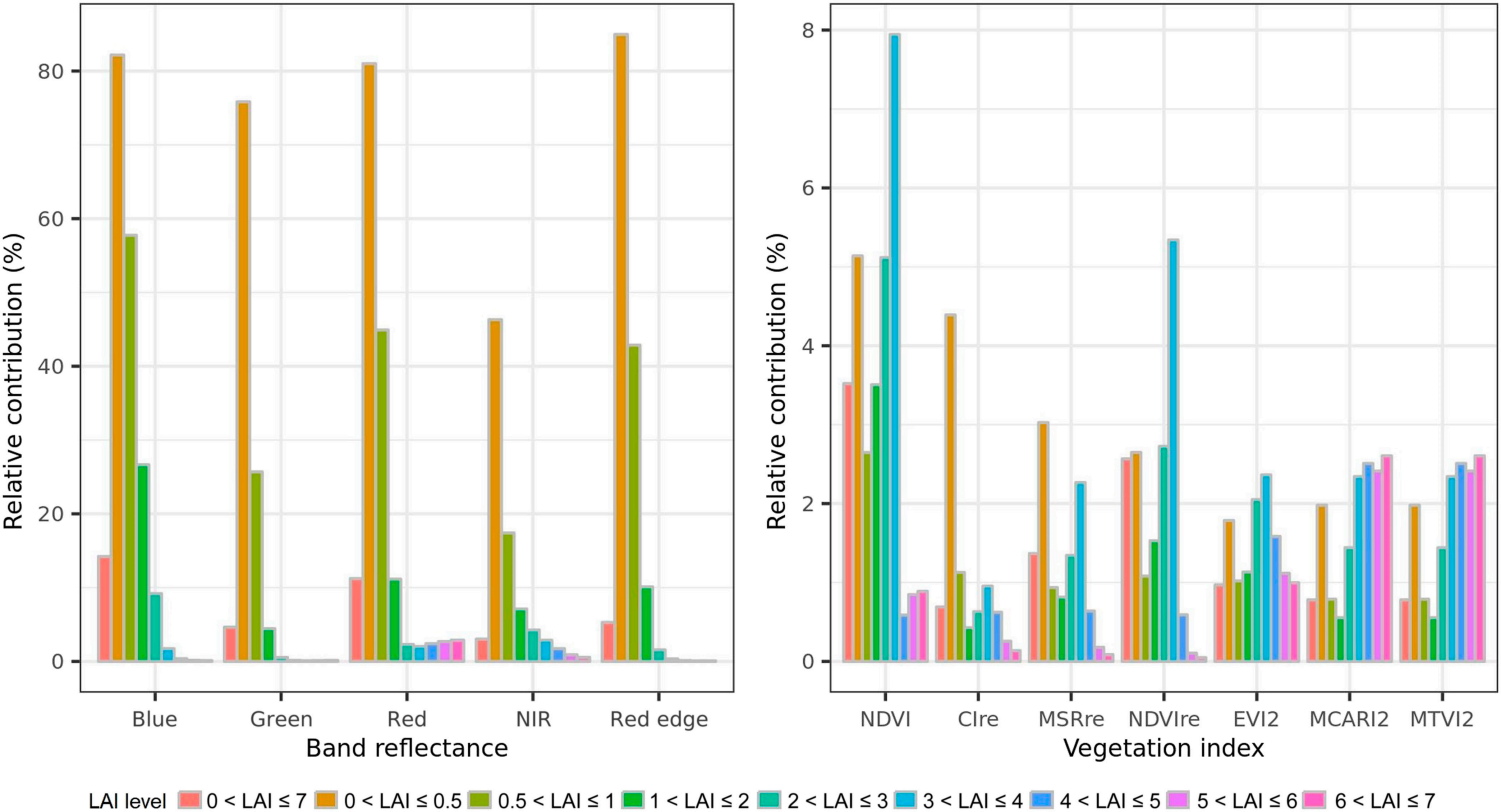
Relative contribution of soil background variation on each band reflectance and vegetation index for varying LAI levels based on an EFAST analysis using data generated by PROSAIL.

Given the defined ranges of input parameters in PROSAIL, a global sensitivity analysis was conducted on the basis of the extended Fourier amplitude sensitivity test (EFAST) [37] to calculate the effects of each input parameter on each band reflectance and each VI for varying LAI levels simulated by PROSAIL. The EFAST was performed using package “sensitivity” (version 1.15.2) in R 3.6.0. Subsequently, the relative contribution of soil background variation on each band reflectance and each VI was calculated from the EFAST results (Fig. 4). Overall, in the full range of LAI (0 < LAI < 7), the reflectance of blue and red bands was more sensitive to variations in soil reflectance than the other 3 bands (i.e., green, NIR, and red edge). The sensitivities of these 3 bands were similar to that of NDVI, while the other 6 VIs were less sensitive than NDVI to soil reflectance variations. The high impacts of soil reflectance variation on canopy reflectance for the 5 bands sharply dropped with increasing LAI for LAI < 2, and its effects were negligible for LAI > 3. The effects of soil reflectance variation on these VIs were relatively small and stable, especially for MSRre, EVI2, MCARI2, and MTVI2 (relative contribution within 3% under all LAI levels).

For strategy 2, 7 sets of canopy-spectral inputs were considered to combine band reflectance and VIs based on sensitivity results, i.e., Ref, RefVI, VI, VIc1, VIc2, VIc3, and VIc4. The “Ref” corresponded to the reflectance of the 5 bands in RedEdge camera. The “RefVI” corresponded to the reflectance of the 5 bands in addition to 7 VIs. The “VI” corresponded to the 7 VIs. The NDVI, NDVIre, CIre, and MSRre were excluded from the 7 VIs step by step to determine the most effective combination of VIs for LAI predictions, resulting in another 4 input sets: VIc1 (i.e., CIre, MSRre, NDVIre, EVI2, MCARI2, and MTVI2), VIc2 (i.e., CIre, MSRre, EVI2, MCARI2, and MTVI2), VIc3 (i.e., MSRre, EVI2, MCARI2, and MTVI2), and VIc4 (i.e., EVI2, MCARI2, and MTVI2). The lower number of features simplifies a predictor and reduces possible combinations leading to a same output, which in turn will reduce the prediction uncertainties. Therefore, we intended to choose a predictor with lower number of features on the basis of maintaining good prediction as the others.

#### The 21 RFR models with and without adoption of any of 2 strategies

On the basis of different combinations of 2 strategies, 21 synthetic training sets (with 30,000 samples each) were generated in total (Table 2). This resulted in 21 different RFR models being considered. The RFR model was named by the soil background and the input set that were used in the training set; for example, model “defaultSingle.Ref” represents the model trained over the synthetic dataset with the “defaultSingle” soil background and the “Ref” canopy-spectral input set.

**Table 2.** Combinations of strategy 1 and strategy 2 used to generate the synthetic training sets. Strategy 1 stands for different sets of training soil background. Strategy 2 stands for different sets of canopy-spectral inputs.

No.	Strategy 1	Strategy 2	No.	Strategy 1	Strategy 2
1	defaultSingle	Ref	12	defaultMulti2	VIc1
2	defaultMulti1	Ref	13	defaultSingle	VIc2
3	defaultMulti2	Ref	14	defaultMulti1	VIc2
4	defaultSingle	RefVI	15	defaultMulti2	VIc2
5	defaultMulti1	RefVI	16	defaultSingle	VIc3
6	defaultMulti2	RefVI	17	defaultMulti1	VIc3
7	defaultSingle	VI	18	defaultMulti2	VIc3
8	defaultMulti1	VI	19	defaultSingle	VIc4
9	defaultMulti2	VI	20	defaultMulti1	VIc4
10	defaultSingle	VIc1	21	defaultMulti2	VIc4
11	defaultMulti1	VIc1			

Notes: “Strategy 1” considers 3 sets of soil background: defaultSingle, a unique soil background including singl esoil reflectance equal to sim2; defaultMulti1, a composite soil background including multiple soil reflectance in the space between sim1 and sim2; defaultMulti2, a composite soil background including multiple soil reflectance in the space between sim1 and sim3. The soil reflectance of sim1, sim2, and sim3 refers to Fig. 3. “Strategy 2” considers 7 sets of canopy-spectral inputs: Ref, including 5 bands (i.e., blue, green, red, NIR, and red edge); RefVI, including 5 bands and 7 VIs (i.e., blue, green, red, NIR, and red edge, as well as NDVI, CIre, MSRre, NDVIre, EVI2, MCARI2, and MTVI2); VI, including 7 VIs (i.e., NDVI, CIre, MSRre, NDVIre, EVI2, MCARI2, and MTVI2); VIc1, including 6 VIs (i.e., CIre, MSRre, NDVIre, EVI2, MCARI2, and MTVI2); VIc2, including 5 VIs (i.e., CIre, MSRre, EVI2, MCARI2, and MTVI2); VIc3, including 4 VIs (i.e., MSRre, EVI2, MCARI2, and MTVI2); and VIc4, including 3 VIs (i.e., EVI2, MCARI2, and MTVI2).

### Evaluating prediction accuracy of RFR models

A series of statistical metrics were used to evaluate the RFR model’s performance from 3 aspects: correlation, fitness, and bias. The Pearson correlation coefficient (*r*) measures the correlation between the observation and its prediction, which is useful to evaluate the degree to which the movement of observed (or known) LAI is captured in predicted LAI, especially in predicting seasonal dynamics of LAI. The determination coefficient (*R*^2^) measures the proportion of the variance in observed (or known) LAI explained by predicted LAI in the linear regression setting, which is suitable to account for the ability of the RFR model to predict LAI for a wide range of conditions. Both root mean square error (RMSE) and RRMSE (a ratio of RMSE divided by the mean of observed (or known) LAI) evaluate the prediction bias of the RFR model, measuring the average absolute and relative error between the known (or observed) LAI and its prediction estimated with the RFR model, respectively. The empirical cumulative density distribution (ECDF) of prediction bias (i.e., the difference of the observed LAI subtracted from the predicted LAI) as well as the derived percentage of samples overestimated (POE) and mean bias error (MBE) were also calculated for bias uncertainty analysis. The POE is a ratio of the number of samples with positive prediction bias divided by the total sample number, presenting in percentage format by times 100. All metrics were calculated in R 3.6.0.

#### Validation on synthetic data generated with new test soils

The soil reflectance of 10 soil samples from the Biomes of Australian Soil Environment (BASE) project [38] was selected on the basis of K-means clustering analysis to represent soil reflectance variations in Australia. The BASE soil reflectance data (695 soil samples in total) were clustered into 10 clusters and then the soil reflectance closest to each of the cluster center was chosen, resulting in 10 selected soil reflectance used in this study. The BASE soil reflectance data are available online (https://zenodo.org/record/6265730). These soil samples vary in color (from dark to white) and texture (different ratios of sand, silt, and clay) (Table S1), and detailed soil attributes are available at the BASE database (https://data.bioplatforms.com/organization/australian-microbiome). The soil reflectance profile (from 350 to 2,500 nm with 1-nm interval) of each soil samples was measured with an ASD spectrometer (PaNalytic, Boulder, Colorado, USA) after a standard preprocessing (e.g., grinding, drying, and preparing on standard plates) in the laboratory [39]. For this study, we focused on representing the possible domain of soil reflectance using a few soil types. Thus, the selection is conducted on the basis of the soil reflectance rather than soil attributes, because different combinations of attributes will result in the same reflectance. Soil10 was selected as an extreme case, although few crops grow on such white and sandy soils. In the current study, the soil reflectance of the 10 selected BASE soils was used to generate synthetic test set characterized by a reflectance spectrum in the range of 400 to 2,500 nm (i.e., soil1 to soil10 in Fig. 3). To evaluate the model’s simulation performance on “new” soils (i.e., not used during the training) without considering the gap between simulated and measured reflectance caused by uncertainties related to model simplification and measurement error, each RFR model was tested on 10 synthetic test sets varying in soils. The 70 test sets (7 canopy-spectral input sets × 10 test soils) were generated in the same way to synthetic training sets with the same canopy-spectral input sets but different soil backgrounds, and each test set has 10,000 samples.

#### Validation on augmented data for early growth stages under different soil backgrounds

**Fig. 5.**
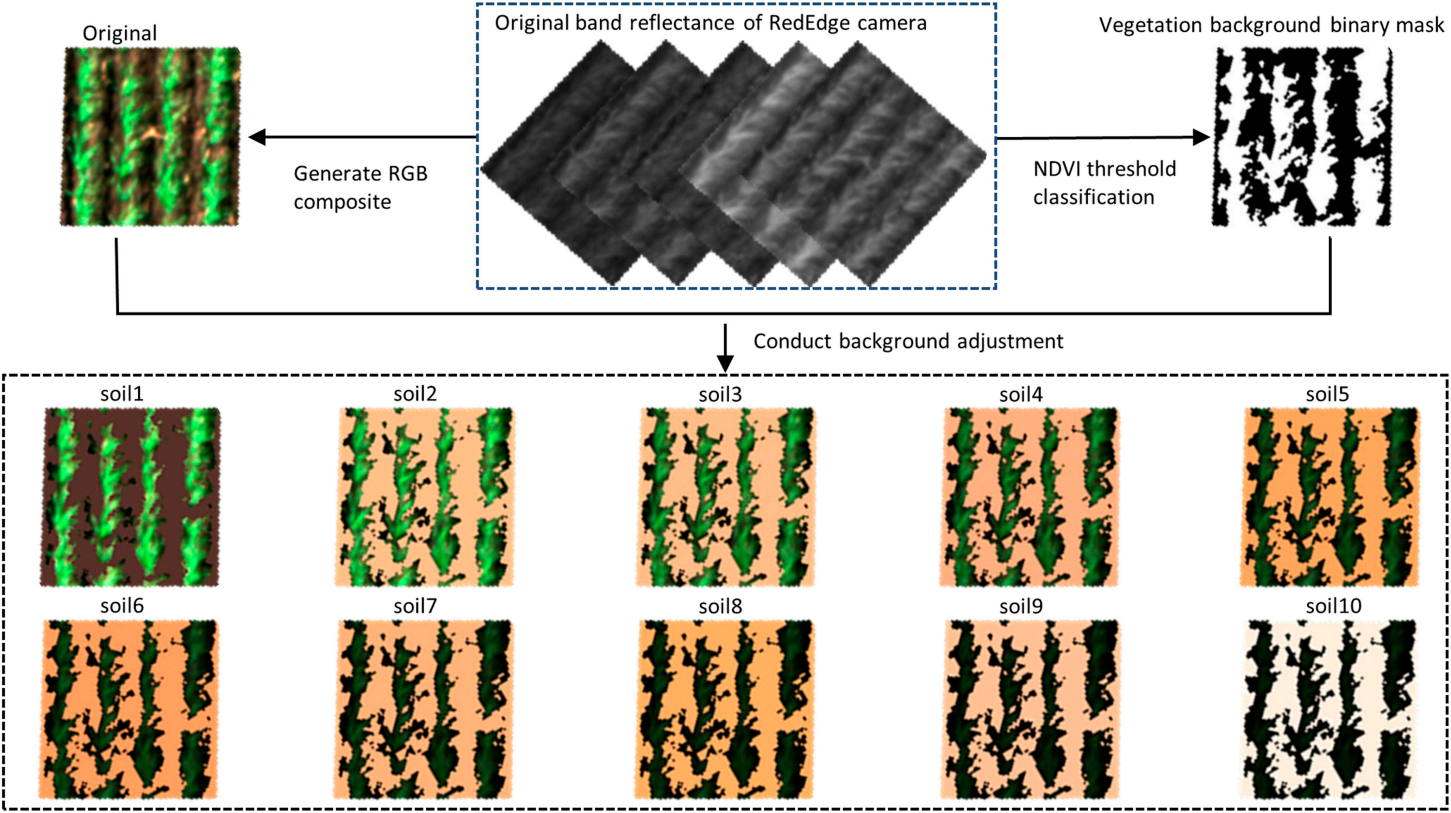
Schematics of using a background adjustment method to generate augmented images presented in RGB composite format. Only a 1-m^2^ area of interest (AOI) was clipped from the field map for presentation to simplify the illustration. The NDVI threshold classification was applied on the NDVI map generated from 2 reflectance bands (i.e., red and NIR) to generate the vegetation background binary mask. This binary mask was used to locate the background pixels to conduct background adjustment on each band reflectance map. Three bands (i.e., red, green, and blue) were composite to create the RGB images to visualize the canopies with and without background adjustment. The “original” has the actual soil background of the AOI. The “soil1” to “soil10” have the soil backgrounds corresponding to the 10 test soils (Fig. 3 and Table S1). The color of these RGB composite images is a false color (or pseudo-color).

In Exp16, all wheat canopies were at seedling stage on June 8 (DAS = 18; flight height of 20 m) when the first flight was conducted. Destructive quadrat harvests were conducted for 84 selected plots on the same date to obtain ground-truth LAI (i.e., observed LAI). At this stage, the LAI was not more than 0.3 m^2^ m^−2^, and a high proportion of soil background was exposed in the UAV-based multispectral images. To evaluate the RFR model’s practical performance for different soil backgrounds, we used the background correction method proposed by Chen et al. [8] (renamed as the “background adjustment” method in the current study) to generate augmented multispectral images under different soil backgrounds. As so, the augmented image does not need to perfectly represent the actual image, as long as this method can create the variability of reflectance images obtained from different soil backgrounds. This was done by replacing the original band value of background pixels with the corresponding soil reflectance of the 10 test soils but keeping the values of vegetation pixels consistent (Fig. 5). Different to the original image with local variation among background pixels, background pixels on augmented image have exactly the same value to the test soil used for background adjustment. The background vegetation classification was realized with an NDVI threshold method, and details can be found in [8]. The red, green, and blue bands of the MicaSense RedEdge camera were composited to generate the RGB image to visualize the augmented images with background adjustment (Fig. 5). The color of these narrow-band RGB composite images is a false color (or pseudo-color) instead of a true color, so they look different from images captured with an RGB camera or what we see with our eyes. Each RFR model was then evaluated on the 84 studied plots with 11 different soil backgrounds (1 original background from UAV and 10 test soil backgrounds presented in Fig. 3).

#### Validation on experimental data in the whole growing season

The RFR models were further tested on all experimental data collected in 2 field experiments to evaluate the prediction accuracy of (a) LAI at different growing stages, (b) the spatiotemporal variation of LAI across the growing season, and (c) the dynamics of genotype-specific LAI along growing season and related heritability under different treatments. On the basis of analysis for experimental data described above, there were 11 (in Exp16) and 4 (in Exp19) observed LAI for each studied plot in the whole growing season. The observed LAI and their predicted values were reorganized on the basis of phenology to evaluate the model’s performance in predicting LAI at different growth stages. On the basis of UAV-based phenotyping dates, the spatiotemporal variation of observed and predicted LAI was mapped in sequence across growing season. In addition, the best linear unbiased prediction (BLUP) model was applied to fit the genotype-specific predictions of LAI (both observed and predicted), which were then used to evaluate model’s performance in predicting the seasonal dynamics of LAI for each treatment varying in genotype, plant density, and water–nitrogen management. According to BLUP analysis results, related heritability and variance components were calculated for each date under different treatments. The BLUP analysis was conducted with the free R package, statgenHTP, available online (https://biometris.github.io/statgenHTP).

## Results

### Model simulation performance on LAI prediction tested on independent synthetic data

The RFR models trained on the synthetic training set were first tested on the unseen synthetic test sets to evaluate their simulation performance. In the VNIR range of 400 to 900 nm, the soil reflectance of soil5 is nearest to that of the defaultSingle soil background; and the reflectance of test soils differs increasingly to that of the defaultSingle soil background from soil5 to soil1 and from soil5 to soil10 (Fig. 3). In the same range of VNIR, the reflectance of the first 5 test soils (i.e., soil1 to soil5) are within the domain of the defaultMulti1 soil background, while all test soils (except for soil10) are inside the domain of the defaultMulti2 soil background (Fig. 3).

**Fig. 6.**
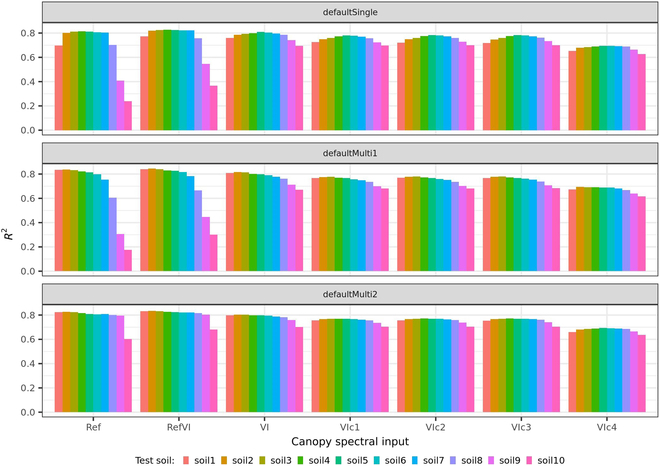
Simulation performance of RFR models for predicting LAI in the range of 0 to 7 m^2^ m^−2^. These RFR models varied in training soil backgrounds (i.e., defaultSingle, defaultMulti1, and defaultMulti2) or/and canopy-spectral input types (i.e., Ref, RefVI, VI, VIc1, VIc2, VIc3, and VIc4). Each RFR model was tested on 10 synthetic test sets corresponding to a specific soil background (i.e., soil1 to soil10).

The baseline model defaultSingle.Ref achieved a high *R*^2^ of 0.8 when tested on test sets with similar soil reflectance as defaultSingle (i.e., sim2) after mean–standard deviation normalization (i.e., soil2 to soil7). The *R*^2^ dropped to 0.7 (soil1 and soil8), 0.4 (soil9), and 0.2 (soil10) (Fig. 6) as the difference of soil reflectance between defaultSingle soil background and test soil background increased (Fig. 3).

For RFR models using Ref (i.e., the 5 bands; see Table 2) as canopy-spectral inputs, broadening the reflectance domain of the training soil background can improve model’s robustness for LAI prediction in different soil backgrounds. Compared with the defaultSingle.Ref model, the defaultMulti1.Ref model obtained better performance when tested on soil1 (increased *R*^2^ from 0.7 to 0.8) but did not improve the performance when tested on soil8 to soil10, while the defaultmulti2.Ref model achieved similarly good performance (*R*^2^ = 0.8) when tested on soil1 to soil9 and less accurate prediction (*R*^2^ = 0.6) when tested on soil10 (Fig. 6).

For RFR models trained over the synthetic training set with the defaultSingle soil background (Fig. 6), improving canopy-spectral inputs reduced the model’s sensitivity to changes in soil background for LAI prediction. The defaultSingle.RefVI model slightly improved *R*^2^ when tested on soil backgrounds (i.e., soil1, soil8, soil9, and soil10), which were poorly predicted by the defaultSingle.Ref model (Fig. 6). All RFR models using input sets that were only consisting of VIs (i.e., VI, VIc1, VIc2, VIc3, and VIc4) had a similar accuracy for LAI prediction for different test soil backgrounds, with a slightly higher accuracy for soils similar to the defaultSingle soil background. The main difference of prediction accuracy between these VI-based models lay in the magnitude rather than the pattern when tested on different soil backgrounds.

Compared with broadening the reflectance domain of training soil background (Fig. 6), improving canopy-spectral inputs allowed more stable LAI prediction across soil backgrounds, especially when tested on soil background highly dissimilar to the training soil background. After improving inputs, adjusting the training soil background further resulted in a slight improvement of prediction accuracy for all tested soils. Overall, 3 models (i.e., defaultMulti2.VIc1, defaultMulti2.VIc2, and defaultMulti2.VIc3) stably achieved reliable LAI estimation for all test soil backgrounds.

The simulation performance was further evaluated for 8 LAI levels: 0 < LAI ≤ 0.5, 0.5 < LAI ≤ 1, 1 < LAI ≤ 2, 2 < LAI ≤ 3, 3 < LAI ≤ 4, 4 < LAI ≤ 5, 5 < LAI ≤ 6, and 6 < LAI ≤ 7 (Fig. S1). As expected, for LAI < 2, both Ref-based and RefVI-based models achieved apparently different prediction accuracy when tested on different soil backgrounds not limited to extreme soils (i.e., soil1, soil8, soil9, and soil10), with RMSE changing in a wide range of 0 to 5 m^2^ m^−2^ (Fig. S1). This was likely due to variations in soil backgrounds presenting a dominant effect on the 5 band reflectance (Fig. 4). Compared with performance of Ref-based and RefVI-based models, the VI-based models performed better across soil types, resulting in a stable prediction accuracy (similar RMSE) for LAI < 2 under different soil backgrounds. Among these VI-based models, further analysis indicated that 3 canopy-spectral input sets (i.e., VIc1, VIc2, and VIc3) were less sensitive to soil variation than the other 2 (i.e., VI and VIc4) across LAI levels (Fig. S2).

**Fig. 7.**
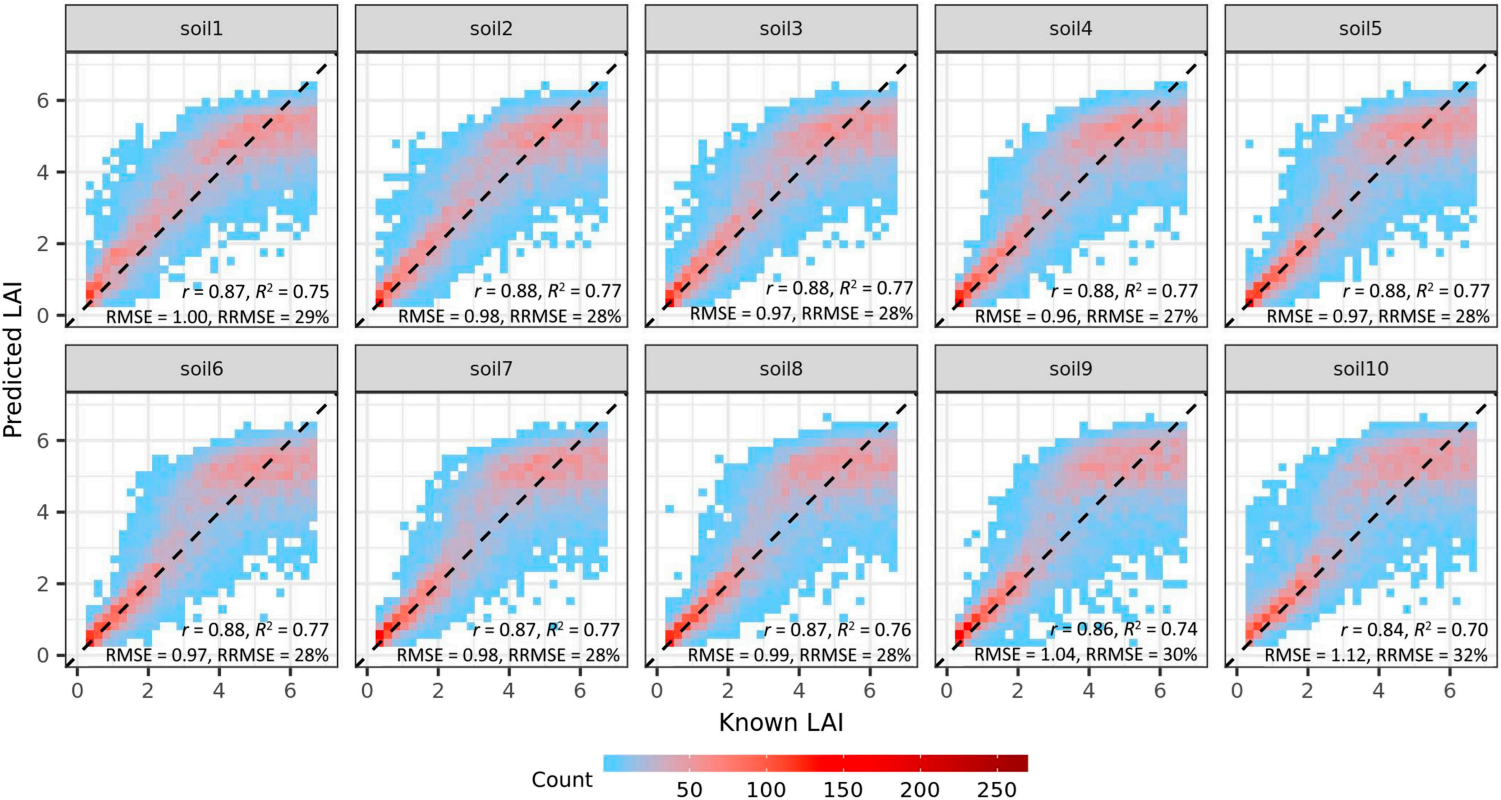
Known LAI against predicted LAI on different synthetic test sets (*n* = 10,000). These synthetic test sets were varied in soil backgrounds (i.e., soil1 to soil10). The predicted LAI was retrieved with the best RFR model (defaultMulti2.VIc3). The known LAI corresponds to LAI values used as inputs in PROSAIL to generate the synthetic datasets.

The defaultMulti2.VIc3 model was selected for further evaluation as (a) the VIc3 input was most stable across soils, especially for extreme soils, although it was slightly more sensitive than VIc1 and VIc2 in general (Fig. S2), and (b) the number of input variables in VIc3 (4 variables) was less than in VIc1 (6 variables) and VIc2 (5 variables). Overall, the defaultMulti2.VIc3 model can achieve similarly good estimation accuracy of LAI for different soil backgrounds, with *r* of 0.84 to 0.88, *R*^2^ of 0.70 to 0.77, RMSE of 0.96 to 1.12 m^2^ m^−2^, and RRMSE of 27% to 32%, but tended to overestimate LAI for 2 < LAI < 5 and underestimate LAI for LAI > 5 irrespective of the soil background tested (Fig. 7).

### Predicting LAI on experimental and augmented data at early growth stage for different soil backgrounds

The established RFR models trained over synthetic training sets were tested on experimental and augmented data for different soil backgrounds. The augmented data were generated from the original experimental data collected at the early growth stage (here, seedling growth with LAI less than 0.3 m^2^ m^−2^) via background soil adjustment as described above. The performance of 4 models was compared to investigate improvements on LAI prediction contributed from 2 proposed strategies (i.e., broadening reflectance domain of the training soil background and improving canopy-spectral inputs). The 4 models (i.e., defaultSingle.Ref, defaultMulti2.Ref, defaultSingle.VIc3, and defaultMulti2.VIc3) were combined from the baseline and best set of training soil background as well as canopy-spectral inputs. The performance comparison of these models was presented in Table 3 and Fig. S3.

**Table 3.** Model prediction accuracy for low LAI (0.01 < LAI < 0.22) in different soil backgrounds. The model accuracy was characterized with root mean square error (RMSE; m^2^ m^−2^) and relative RMSE (RRMSE). The predicted LAI was retrieved using different RFR models (i.e., defaultSingle.Ref, defaultMulti2.Ref, defaultSingle.VIc3, and defaultMulti2.VIc3) from experimental and augmented multispectral data for different soil backgrounds at the early growth stage (i.e., seedling growth stage; DAS = 18 in Exp16). The “original” stands for the experimental multispectral images with an original background, while “soil1” to “soil10” represent the augmented multispectral images with test soil backgrounds.

Model prediction accuracy (RMSE/RRMSE)				
Test soil background	defaultSingle.Ref	defaultMulti2.Ref	defaultSingle.VIc3	defaultMulti2.VIc3
Original	2.25/2,317%	0.13/117%	0.15/134%	0.23/212%
soil1	2.38/2,190%	0.12/114%	0.12/108%	0.19/173%
soil2	1.43/1,315%	0.12/113%	0.07/61%	0.09/66%
soil3	1.22/1,122%	0.14/126%	0.08/77%	0.10/88%
soil4	1.00/923%	0.25/227%	0.13/117%	0.10/94%
soil5	0.85/780%	0.45/417%	0.05/42%	0.08/76%
soil6	0.79/730%	0.52/482%	0.04/40%	0.05/48%
soil7	0.50/472%	0.31/288%	0.06/58%	0.04/35%
soil8	0.66/607%	0.43/400%	0.05/50%	0.12/111%
soil9	2.35/2,162%	0.20/183%	0.06/77%	0.03/32%
soil10	2.99/2,757%	0.92/843%	0.22/200%	0.25/233%

For the defaultSingle.Ref model, substantial differences existed in prediction accuracy (RMSE changing from 0.5 to 2.99 m^2^ m^−2^) when tested on different soil backgrounds (Table 3). Consistent to simulation performance, the defaultSingle.Ref achieved higher accuracy in predicting LAI for soil backgrounds with a high similarity to the training soil background. For example, the defaultSingle.Ref model achieved more accurate LAI prediction when tested on backgrounds from soil4 to soil7 (with RMSE within 1 m^2^ m^−2^ and maximum bias within 1.5 m^2^ m^−2^) than the other backgrounds (with RMSE over 1 m^2^ m^−2^ and maximum bias up to 5 m^2^ m^−2^) (Table 3 and Fig. S3). The soil reflectance of the original background was closest to the soil reflectance of soil1 among the 10 test soils (Fig. 3), so the model’s prediction accuracy was similar for these 2 backgrounds (Table 3).

From the 2 proposed strategies to improve the RFR model, strategy 2 (i.e., improving canopy-spectral inputs) was more effective at predicting LAI across soil types than strategy 1 (i.e., broadening reflectance domain for training soil background). Compared with defaultSingle.Ref, the defaultMulti2.Ref model reduced the range of RMSE from 0.5–2.99 to 0.12–0.59 m^2^ m^−2^, while the defaultSingle.VIc3 model reduced to a smaller range of 0.04 to 0.22 m^2^ m^−2^ (Table 3). After improving canopy-spectral inputs used in RFR models, broadening the reflectance domain for training soil background appeared not to further improve prediction accuracy, with RMSE from the defaultMulti2.VIc3 model ranging from 0.03 to 0.25 m^2^ m^−2^ (Table 3). Although the defaultSingle.VIc3 model appeared to perform slightly better than the defaultMult2.VIc3 model at this seedling growth stage for LAI < 0.3, the latter was further tested in the following sections as it appeared more stable across LAI levels in simulation analysis.

### Predicting LAI at different growth stages for the whole growing season

The baseline model (i.e., defaultSingle.Ref) and the model with the best simulation performance (i.e., defaultMulti2.VIc3) were further tested on experimental data collected in 2 field experiments to evaluate the model’s practical performance in predicting LAI at different growth stages across the growing season. Compared with the defaultSingle.Ref model, the improvement of LAI prediction from the defaultMulti2.VIc3 model mainly occurred in LAI < 2 irrespective of growth stage (Fig. S4), which was consistent with the results from teh simulation analysis.

**Fig. 8.**
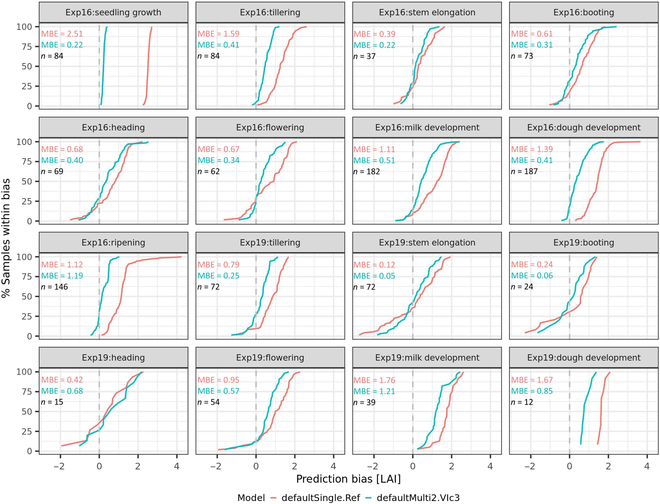
Empirical cumulative density distribution (ECDF) for LAI prediction bias at different growth stages. The bias indicates the difference of observed LAI subtracted from LAI predicted by RFR models (i.e., defaultSingle.Ref and defaultMulti2.VIc3) from real multispectral data captured at different growth stages (i.e., seedling growth, tillering, stem elongation, booting, heading, flowering, milk development, dough development, and ripening) in 2 field experiments (i.e., Exp16 and Exp19).

For the defaultSingle.Ref model, the calculated ECDF presented a plausible seasonal changing pattern in both proportion (expressed in POE) and magnitude (expressed in MBE) of prediction bias: decreasing from seedling growth to stem elongation, maintaining a relative low value until flowering, and increasing from flowering to ripening (Fig. 8). This seasonal changing pattern synchronized with seasonal changes in the fraction of non-green background, which decreased before it started to increase along with vegetation development and senescence (Fig. 2). Compared with the defaultSingle.Ref model, the defaultMulti2.VIc3 model presented a similar changing pattern in the proportion of overestimation but a much smaller magnitude of overestimation, especially at early (i.e., seedling growth and tillering) and late stages (i.e., milk development, dough development, and ripening) (Fig. 8) of which there was a high percentage of samples with observed LAI less than 2 m^2^ m^−2^ (Fig. S4). The substantial improvement in prediction at early and late stages indicated that the defaultMuti2.VIc3 model was unaffected by changes in soil and dead leaf backgrounds.

**Fig. 9.**
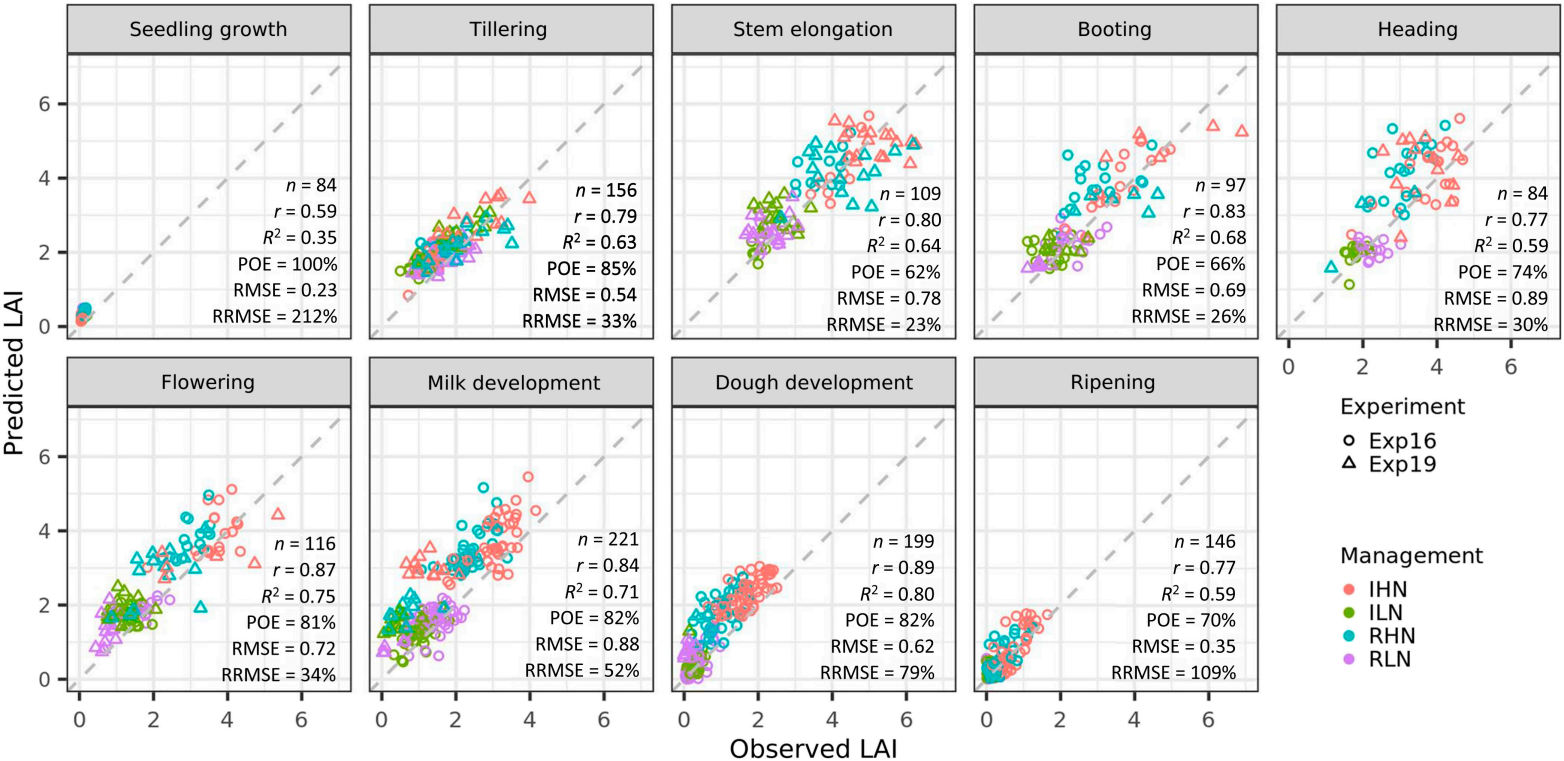
Observed LAI against predicted LAI at different growth stages in 2 field experiments with different genotypes and management practices. Predicted LAI was obtained with the best RFR model (i.e., defaultMulti2.VIc3) that used real multispectral data captured at different growth stages under different water–nitrogen managements (i.e., IHN, ILN, RHN, and RLN) in 2 field experiments (i.e., Exp16 and Exp19).

The defaultMulti2.VIc3 model provided prediction of LAI strongly correlated to observed LAI (*r* of 0.77 to 89) during growing season except at the seedling growth stage (*r* = 0.59) (Fig. 9). The model tended to overestimate LAI (POE of 62% to 100%), but the possibility of overestimation reduced at stem elongation (POE = 62%) and booting stage (POE = 66%) as LAI approached its peak value. As expected, the RMSE (0.23 to 0.62 m^2^ m^−2^) was smaller for sparse canopies at early (i.e., seedling growth and tillering) and late stages (i.e., dough development and ripening) but was larger for dense canopies from stem elongation to milk development (RMSE of 0.69 to 0.89 m^2^ m^−2^). By contrast, the RRMSE was larger for sparse canopies and smaller for dense canopies.

### Mapping the spatiotemporal variation of LAI across growing season

**Fig. 10.**
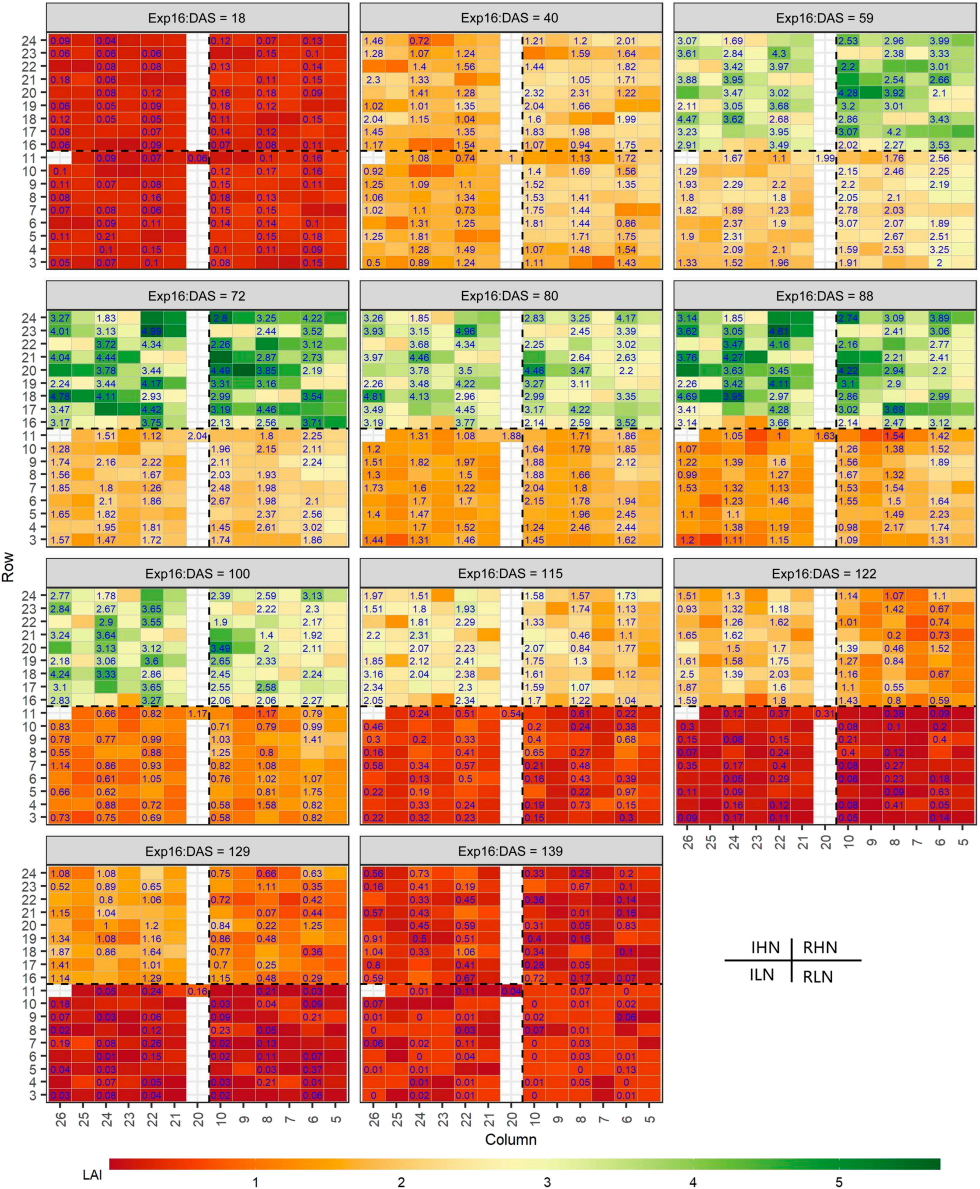
Predicted LAI for different phenotyping dates in Exp16. The predicted LAI was retrieved with the best RFR model (defaultMulti2.VIc3) using experimental multispectral data captured with the UAV platform. The plots with numbers at the top indicate those designed for UAV-based phenotyping, while the number denotes the observed LAI from the corresponding plots used for destructive harvests. For each subfigure, only 84 plots have observed LAI as biophysical measurements were only conducted in these plots. Rows and columns are used to locate the position of the plot in the field. The 4 blocks correspond to the 4 water–nitrogen treatments, i.e., IHN, ILN, RHN, and RLN.

The defaultMulti2.VIc3 model was used to map the spatiotemporal variation of LAI within/between 4 blocks across growing season for 2 experiments, as shown in Fig. 10 (for Exp16) and Fig. S5 (for Exp19). Within each map, the 4 blocks are corresponding to the 4 water–nitrogen treatments, i.e., IHN (top left), RHN (top right), ILN (bottom left), and RLN (bottom right). Same with the spatiotemporal pattern presented in the observed LAI, the predicted LAI can reliably capture the increasing trend of plot-scale LAI along with time and the water–nitrogen effects on LAI across growing season in both field experiments (Fig. 10 and Fig. S5). At the beginning of the growing season, the differences among treatments were small. The LAI values under high nitrogen treatments (IHN and RHN) were substantially higher than those under low nitrogen treatments (ILN and RLN) despite applying irrigation or not, with tillers developing and leaves expanding. Approaching the end of the season, the difference among treatments diminished as senescence of green parts began to increase. In most situations, the predicted LAI can correctly identify the negative or positive symbol of between-group difference among water–nitrogen treatments at different UAV-based phenotyping dates even under varying sowing densities, although the magnitude of difference of group means might be different (Figs. S6 and S7).

### Predicting the dynamics of genotype-specific LAI and related heritability during the whole growing season

**Fig. 11.**
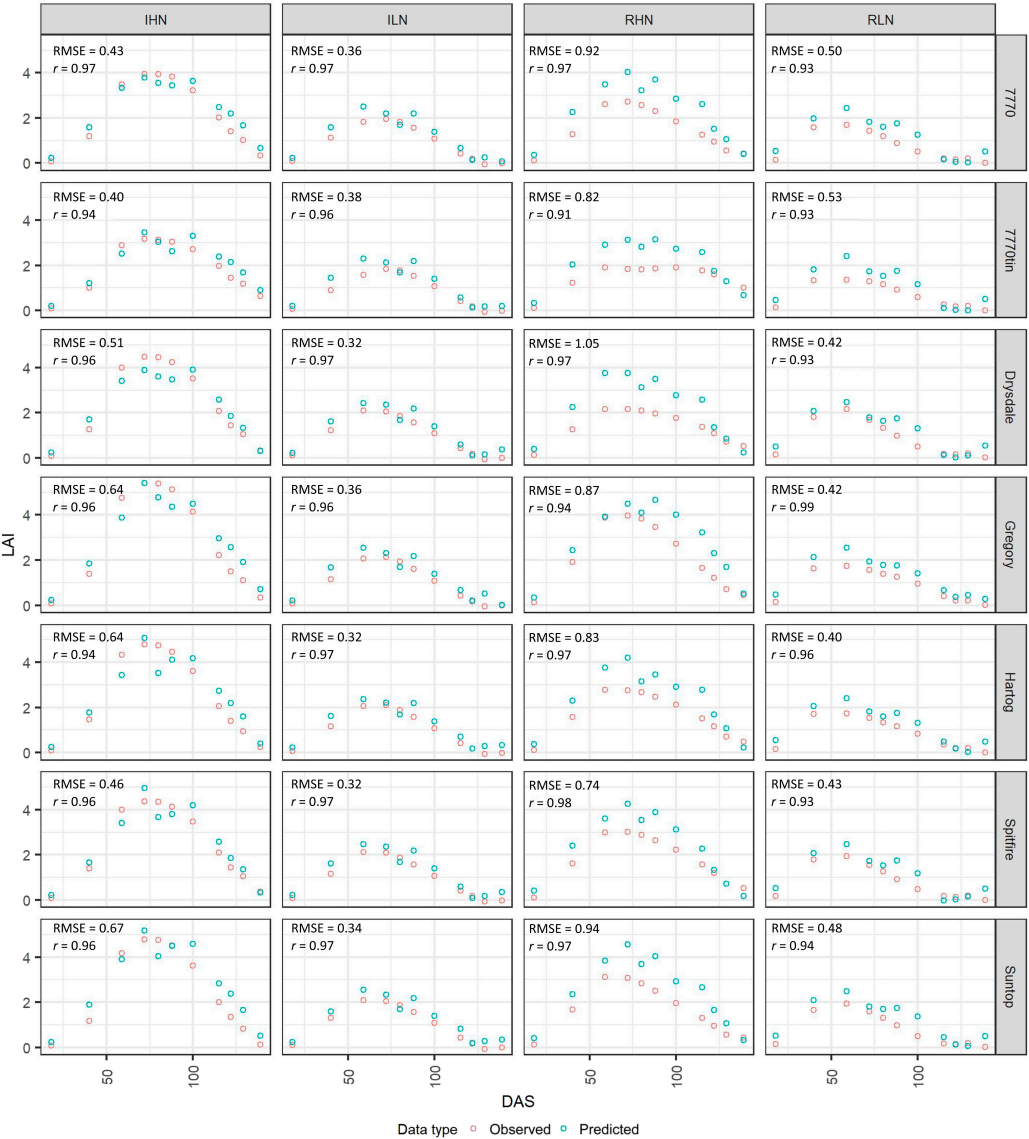
The dynamics of the genotype-specific observed LAI and predicted LAI over the growing season for Exp16. The red symbols indicate the genotype-specific observed LAI obtained from the destructive harvest. The blue symbols correspond to the genotype-specific predicted LAI retrieved with the best RFR model (defaultMulti2.VIc3) using experimental multispectral data captured with the UAV platform. The genotype-specific values of LAI were calculated with the best linear unbiased prediction (BLUP) model. The 4 water–nitrogen treatments include IHN, ILN, RHN, and RLN.

The defaultMulti2.VIc3 model was tested on experimental data to evaluate its ability in predicting the seasonal dynamics of LAI in 2 field experiments. Observed LAI measured from destructive harvests and LAI predictions from the RFR model using UAV-based multispectral images are presented for the different genotypes, plant densities, and irrigation–fertilization managements in Fig. 11 (Exp16) and Fig. S8 (Exp19). Over the growing season, the observed LAI gradually increased before reaching its peak value and then started to decrease with leaf senescence. The difference in LAI dynamics among genotypes and plant densities was relatively small, while the difference among irrigation–fertilization managements was relatively obvious, especially between the high-nitrogen and low-nitrogen treatments.

The genotype-specific value of LAI predicted with defaultMulti2.VIc3 model using multispectral data presented remarkable consistency of the temporal dynamics compared with the observed LAI, with a value of *r* over 0.9 for 49 of 64 treatments in the 2 field experiments (Fig. 11 and Fig. S8). The *r* value of the remaining 15 treatments in Exp19 slightly reduced from 0.82 to 0.89 (Fig. S8). Further analysis for Exp16 indicated that the predicted value can reliably characterize the heritability of 7 genotypes under high nitrogen treatments (i.e., IHN and RHN; Fig. S9). However, the heritability under low nitrogen treatments (i.e., ILN and RLN; Fig. S9) was low from either observed or predicted values because the genotypic variance under ILN and RLN was close to the residual variance (Fig. S10). For Exp19, only the heritability under IHN was well characterized by predicted values (Fig. S11). The inconsistent heritability calculated from observed and predicted LAI under the other treatments was mainly due to (a) the genotypic variance not being large enough than residual variance to distinguish genotypes (Fig. S12), and (b) the defaultMulti2.VIc3 model tended to underestimate high LAI (Figs. 7 and 9).

## Discussion

### Two strategies to improve LAI predictions from UAV-based imagery irrespective of soil backgrounds

This study developed a background-resistant model to predict LAI that used 2 strategies, i.e., an extended reflectance domain of the training soil background and an improved set of canopy-spectral indicators as model inputs. In theory, this background-resistant model can provide accurate LAI prediction for different LAI levels and for diverse soil backgrounds (Fig. 7), because high accuracies were achieved for a predictive model trained and tested with the similar synthetic data across a wide range of LAI [8,9,13]. By contrast, the baseline model (defaultSingle.Ref) made better prediction for some soil backgrounds than others (Fig. 6) depending on the similarity between the training and the test soil backgrounds. Extending the reflectance domain of the training soil background increased the diversity of the training dataset (i.e., changing defaultSingle to defaultMulti1 and to defaultMulti2), potentially making the test set a subset of the overall distribution represented by the training set. This improved the model’s robustness for LAI prediction in different soil backgrounds. Previous studies also used multiple soil reflectance instead of a single soil reflectance to generate synthetic training data when applying predictive models for LAI prediction from satellite images in a large region, but they did not compare the performance of models trained over single and multiple soil reflectance [10,27].

Improving canopy-spectral inputs essentially corresponds to extracting the common features correlated to LAI in different soil backgrounds, leading to a generic relationship between LAI and spectral signals. Our study found that Ref-based and RefVI-based models were able to achieve accurate LAI prediction except for some extreme soils that were highly dissimilar to training soils. By contrast, the VI-based models achieved much more accurate LAI prediction for these extreme soils (i.e., very white and sandy) (Fig. 6), which was consistent with a similar study about comparison of LAI predicted from reflectance or VIs [40]. Although few crops grow in these extreme soils, the consideration on these situations might be worth for vegetation monitor in other disciplines (e.g., ecology). Previous studies indicated that compared to visible-based VIs, red edge-based VIs were less sensitive to canopy structures (e.g., average leaf angle) and were more effective for medium to high LAI estimation [26]. It was also reported that VIs such as EVI2, MCARI2, and MTVI2 were stable across soil and pigment variations and were less prone to saturation at high LAI [1,41]. In addition, we found that the sensitivity to changes in soil background varied across both LAI levels and VIs, with varying sensitivity among LAI levels for NDVI substantially higher than others (Fig. 4). These highlighted the importance of an appropriate combination of VIs to achieve accurate prediction of LAI in diverse soils. Although several models achieved similarly good performance, the defaultMulti2.VIc3 model with fewer input variables was finally selected for detailed analysis in the current study.

### Difference between the simulation and practical performances of the model

It is common for predictive model trained over synthetic data to achieve different or less accurate LAI prediction when tested on real experimental data [13]. The essential reason may come from simplifications embedded in RTMs that result in inherent differences between observed and simulated canopy reflectance under given conditions [19]. Another reason is the difference of soil backgrounds, which is assumed to be pure soil in RTM simulations but was a mixed of soil and plant residues in actual observations in the field. This issue was reported to be effectively addressed by (a) adding noise to synthetic training data [10], (b) conducting background correction on spectral images [8], (c) establishing a relationship between LAI and VI less sensitive to soil variation [1], or (d) developing a background-resistant model as presented in current study. Additionally, the model performance when tested against synthetic data can also be, at times, reduced by the large bias from outliers that were predicted by the model, which may yet have a high overall accuracy [8,12]. For example, in the current study, the defaultMulti2.VIc3 model stably achieved a good prediction of LAI on both synthetic test data with different soil backgrounds (*R*^2^ of 0.70 to 0.77 and RRMSE of 27% to 32%; Fig. 7) and experimental data (*R*^2^ of 0.73 to 0.89 and RRMSE of 41%; Fig. S13). However, the prediction bias on synthetic data was symmetrically distributed in 2 sides of zero, ranging from −5.67 to 4.51 m^2^ m^−2^, while on experimental data, the prediction bias fluctuated in a smaller range (−1.84 to 2.53 m^2^ m^−2^), with more samples overestimated (about 75%) than underestimated (about 25%) (Fig. S13). The outliers might be due to the less-accurate relationships established from the training data containing some unrealistic samples whose combinations of values of crop traits cannot be observed in the real world, and this issue can somehow be addressed by introducing biological constraints in training data as presented by Chen et al. [9].

### Different performances of the model at different growth stages

Most studies estimating LAI based on multispectral imagery were undertaken during the crop vegetative stages to not consider the effects of heads or senescent vegetation. Such studies typically achieve different prediction accuracies for LAI of wheat depending on sites and growth stages [1,8,11,40,42]. During the wheat growing cycle, the fraction of green vegetation (fcover) gradually increases over time and maintains a high value (with the consecutive development of leaves, stems, and heads) before it starts to decrease because of tissue senescence [43]. Accordingly, the opposite seasonal pattern occurs for the fraction of non-green background (1 − fcover). The change over time in the bias of LAI predicted with the baseline RFR model (i.e., defaultSingle.Ref) in this study (Fig. 8) can be explained from (a) the seasonal changing pattern of the fraction of non-green background (i.e., soil and senesced tissues) and (b) effects of background variations on canopy reflectance in the VNIR range at different LAI levels (Fig. 4). The improved RFR model (i.e., defaultMulti2.VIc3) reduced the effects of background variations (Fig. 8) and resulted in relatively stable predictions of LAI across growth stages (Fig. 9), which proved the effectiveness of the proposed strategies in developing a background-resistant model.

In growing crops, the canopy reflectance captured using sensors results from the characteristics of the canopy, which consist of all aboveground components including leaves, stems, and reproductive organs (i.e., heads, ears, and tassels) [44]. The predicted LAI from the real spectral image based on PROSAIL simulations did not correspond to LAI but GAI that included other green organs (e.g., stems and heads) in addition to leaves. As the growing season progresses, there is a temporal shift between the GAI and LAI as described by Duveiller et al. [7]. This explains a quasi-systematic overestimation of “LAI” (i.e., GAI in reality) predicted with the RFR model in this study (Fig. 8). The high proportion of a slight overestimation at early stages (i.e., seedling growth and tillering) was due to ignoring of the stem area in the observed LAI. For stem elongation to booting, effects from the saturation for dense canopy were offset by impacts from the appearance of heads, resulting in a lower proportion of overestimation during growing season. From heading onwards, the occurrence of senescence affected prediction accuracy of LAI through changing the co-distribution of green components. The prediction accuracy could be improved by establishing a transformation between GAI and LAI based on the proportion of stems, leaves, and heads [7]. Because of the simplification of canopy description in 1-dimensional (1D) RTM such as PROSAIL, the hybrid methods based on 1D canopy simulations generally result in underestimation of LAI at a high value as presented in the current study (Fig. 7) and others [8,12]. More realistic reflectance simulations using 3D canopy models could effectively address this underestimation issue as presented by Jiang et al. [45]. These authors also indicated that the effective GAI (GAI_eff_) can be better estimated from canopy reflectance than GAI or LAI.

### A method that captures seasonal changes in LAI

Previous studies have confirmed the temporal consistence of predicted LAI dynamics from multisource data collected from different satellites [10,25]. However, to our knowledge, the quantification of seasonal dynamics of the predicted LAI has not yet been properly characterized and validated at plot scale because of the lack of sufficient measurements for ground truthing. Only a few studies at the field or region scale have used coarse satellite images together with ground measurements [46–48]. The current study investigated model performance in capturing seasonal LAI dynamics at plot scale over a sufficient dataset consisting of 2 growing seasons for different treatments (i.e., genotypes, plant densities, and water–nitrogen managements). The established background-resistant model provided reliable alignment to the observed seasonal changing pattern of genotype-specific values (Fig. 11 and Fig. S8). Importantly, this background-resistant model was sensor-specific and can be established in advance without using any ground calibration. This means that this single ready-to-use model can predict the LAI dynamics at plot scale irrespective of the soil background, thus providing a friendly LAI high-throughput phenotyping method that could potentially benefit in-season phenology identification and crop growth rate assessment [49].

### Limitations and perspectives

The reflectance profiles of the different soils used in the defaultMulti2 training soil background were linearly correlated. Incorporating multiple soil reflectance without linear correlation is possible to further improve the robustness of model performance by increasing the diversity of the soil training set. In addition to the approach used to simulate diverse soil reflectance spectra, some soil reflectance models, such as the Price model [50] and GSV model [51], can be used to create the variability of soil reflectance spectra. Moreover, additional efforts could be put to develop better VIs and VI combinations to further improve the background-resistant model and enhance prediction accuracy for LAI and other traits for other species in the future.

In the practical validation of this study, the prediction was computed from plot reflectance and derived VIs. The plot area (approximate 8.96 m^2^ for Exp16) used for aggregation was similar to the resolution of satellites such as CubeSat (3 m × 3 m). Hence, this method could be used with UAV-based images to produce ground truths of LAI to validate satellite-based estimates. Furthermore, this generic model is theoretically able to produce reliable prediction of wheat LAI directly from high-resolution satellite images given its background-resistant attribute, which should be able to deal with mixed pixels (i.e., aggregation of vegetation and soil in each pixel). The increasing spatial resolution enables a more accurate capture of details in the canopy, but the RTM-based method is not suitable to apply to make prediction directly on pixels, unless the pixel size is big enough to represent a canopy. On the basis of our understanding, the minimum area is 50 cm × 50 cm for wheat canopy. This is the reason that we applied our RFR model trained on PROSAIL simulations on plot mean reflectance to predict plot-scale LAI. To enhance the advantage of images with sub-centimeter resolution, different non-RTM-based approaches were investigated to estimate LAI with pixel values [52]. For utilization of satellite images with big-enough pixels, the spatial resolution did not show a substantial difference of LAI estimation retrieved with linear models regardless of VIs used to build the LAI–VI relationship [53]. However, effects of spatial resolution on LAI estimation retrieved with RTM-based LUT (look-up table) did not reach an agreement [53,54]. That is likely because LUT-based estimation of LAI is based on the similarity between spectral data instead of the relationship between spectral data and LAI. Another study for LAI estimation using machine learning methods indicated that there was no substantial difference between LAI estimations retrieved from satellite imagery-derived canopy spectral information or UAV-based canopy structure information, but prediction accuracy was boosted by integration of spectral and structure information as the integration reduced background soil effect and asymptotic saturation issue to some extent [55].

The proposed background-resistant model is sensor specific, but it can be updated for other sensors. In this case, simulated canopy reflectance needs to be resampled into band reflectance in accordance with the spectral response function of the target sensor. The idea of developing a background-resistant model is not limited to wheat LAI and can be applied to predict other traits and for other species in associated research areas (e.g., breeding, agriculture, ecology, vegetation remote sensing, etc.), provided that appropriate PROSAIL input parameters and effective VIs are defined for the target trait and/or the target species.

### Conclusion

In this study, we applied a hybrid method to establish the predictive model to predict wheat LAI by training RFR models over synthetic data simulated by PROSAIL without any ground calibration. Two strategies (i.e., broadening the reflectance domain of the training soil background and improving canopy-spectral indicators as model inputs) were investigated to reduce the sensitivity of the RFR model to background variation for LAI prediction. Both strategies presented to improve the robustness of prediction accuracy on unseen synthetic data in different soil backgrounds, while improving canopy-spectral inputs appeared to be more effective and robust. The improved RFR model (i.e., defaultMulti2.VIc3), which adopted an extended reflectance domain of training soil background and a soil-insensitive input set with fewer canopy-spectral indicators, was proved to be resistant to background variation on synthetic data. On the basis of validation in field experiments, this defaultMulti2.VIc3 model presented stable prediction accuracy for low LAI retrieved from the original image and augmented images even in extreme soil background. In addition, this model achieved similar prediction accuracy for LAI at different growth stages, with relatively larger RMSE in mid-season from stem elongation to milk development (dense canopies) and smaller RMSE at early and late stages (sparse canopies). Moreover, this model produced predicted LAI strongly correlated to their observations, reliably capturing the seasonal pattern of LAI dynamics under different treatments in terms of genotypes, sowing densities, and water–nitrogen managements. As discussed above, the prediction retrieved from spectral images generally corresponds to GAI (all green parts included) and only represents LAI in the case that green leaves are separated from the other green organs (e.g., stems and heads). This explains a quasi-systematic overestimation of “LAI” (i.e., GAI in reality) predicted with the RFR models in this study. Overall, the good prediction of the defaultMulti2.VIc3 model obtained from synthetic, augmented, and experimental data indicated that a background-resistant model can be established using simulation data. This ready-to-use background-resistant model can enable a stable and accurate GAI prediction from isolated UAV-based multispectral images over wheat growing season for diverse soil backgrounds in field conditions.

## Data Availability

Details and source code of the PROSAIL model used in this study are openly available at http://teledetection.ipgp.jussieu.fr/prosail/. Other data and source code supporting this work are available at UQ eSpace, and a unique DOI (https://doi.org/10.48610/ac9642c) is provided for public access.
