# Supplementary material for: A Generic Model to Estimate Wheat LAI over Growing Season Regardless of the Soil-Type Background
Source: Plant Phenomics. 2023 May 23;5:0055. doi: 10.34133/plantphenomics.0055 (PMC10205590; doi:10.34133/plantphenomics.0055)
Supplement: Supplementary 1 — Figs. S1 to S13. Table S1. [file plantphenomics.0055.f1.docx]

**A generic model to estimate wheat LAI over growing season regardless of the soil-type background**

Qiaomin Chen ^1, 2 *^, Bangyou Zheng ^2^, Karine Chenu ^3^, Scott C. Chapman ^1 *^

**Supplementary materials**

# Supplementary figures


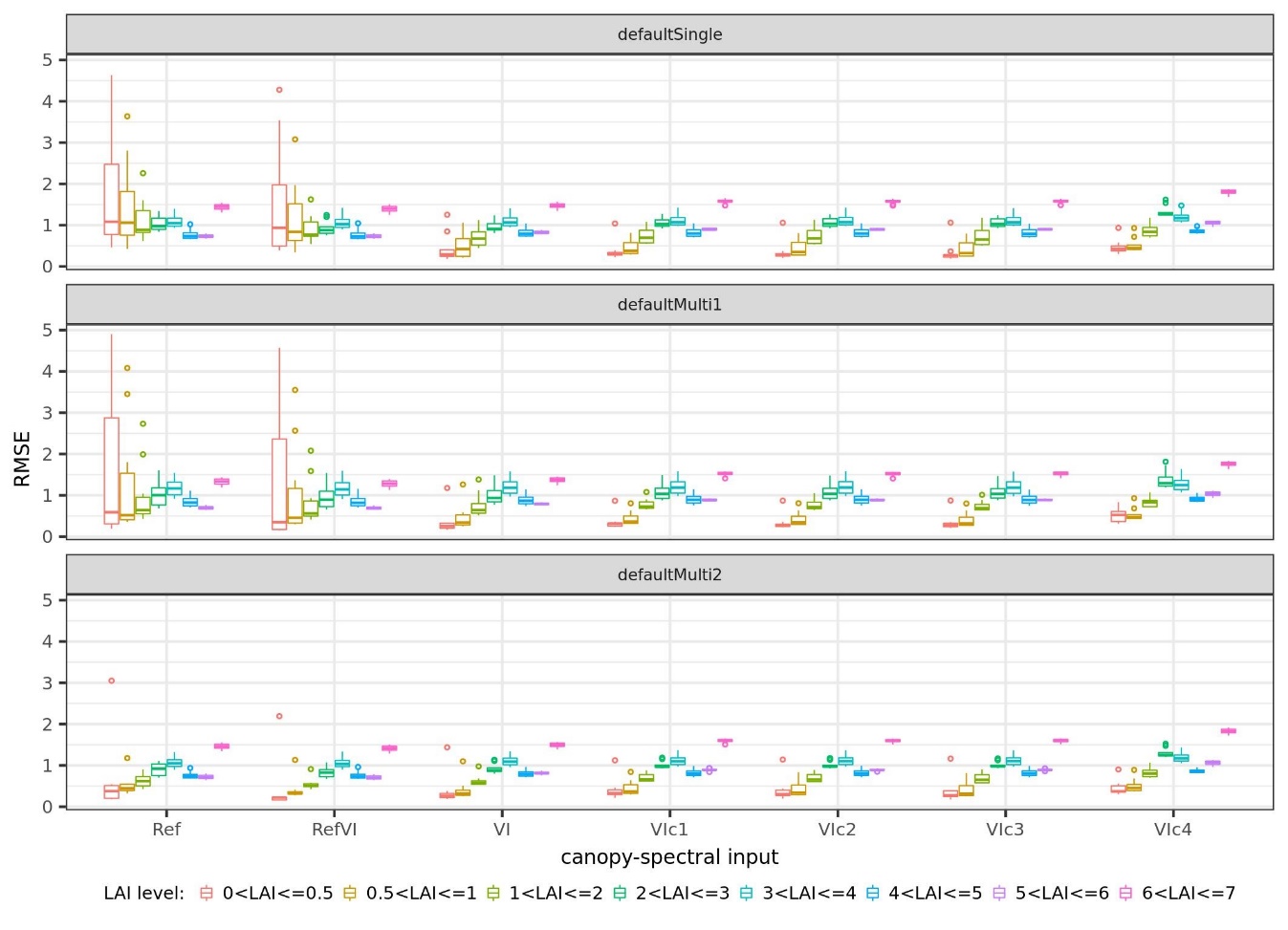


Figure S1 Theoretical performance of RFR models to predict LAI for different LAI levels on synthetic test sets under different soil backgrounds. “defaultSingle”, “defaultMulti1” and “defaultMulti2” indicate different training soil backgrounds.


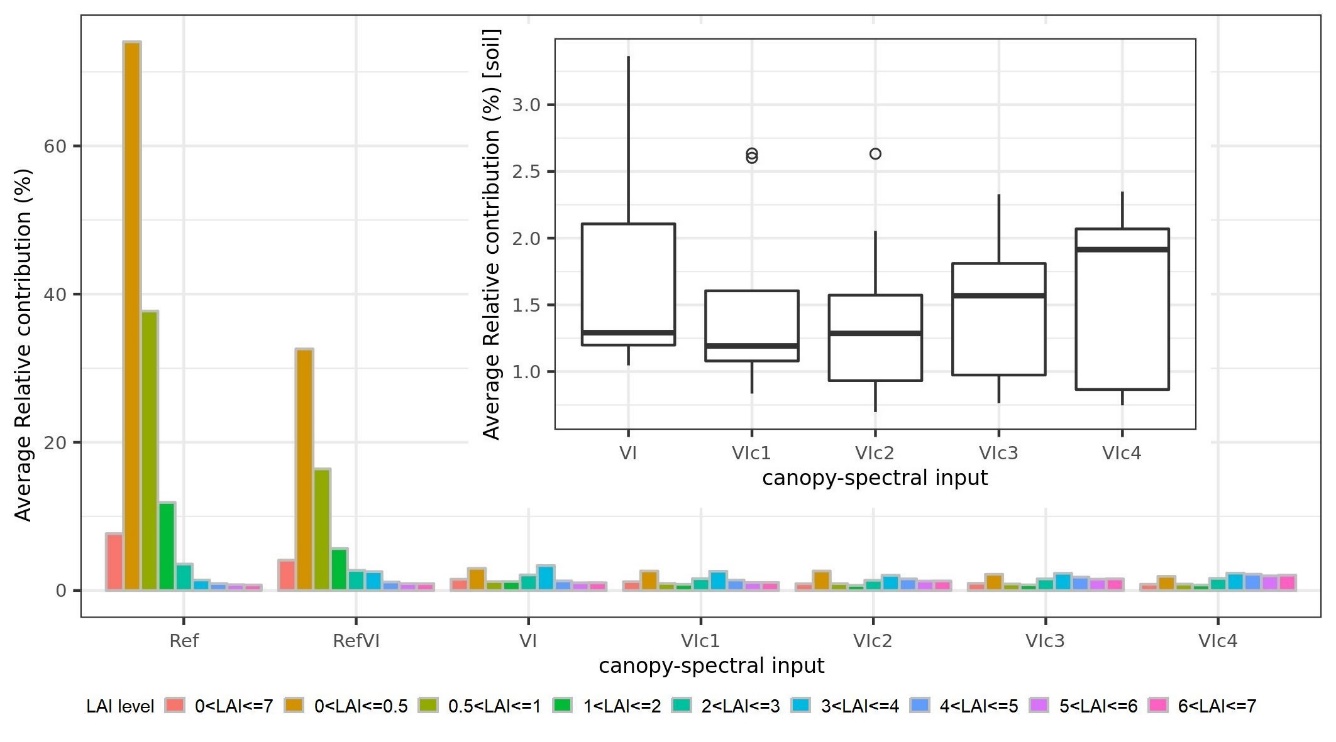


Figure S2 Relative contribution of variation of soil background on each canopy-spectral input type for varying LAI levels based on EFAST analysis using PROSAIL simulations.


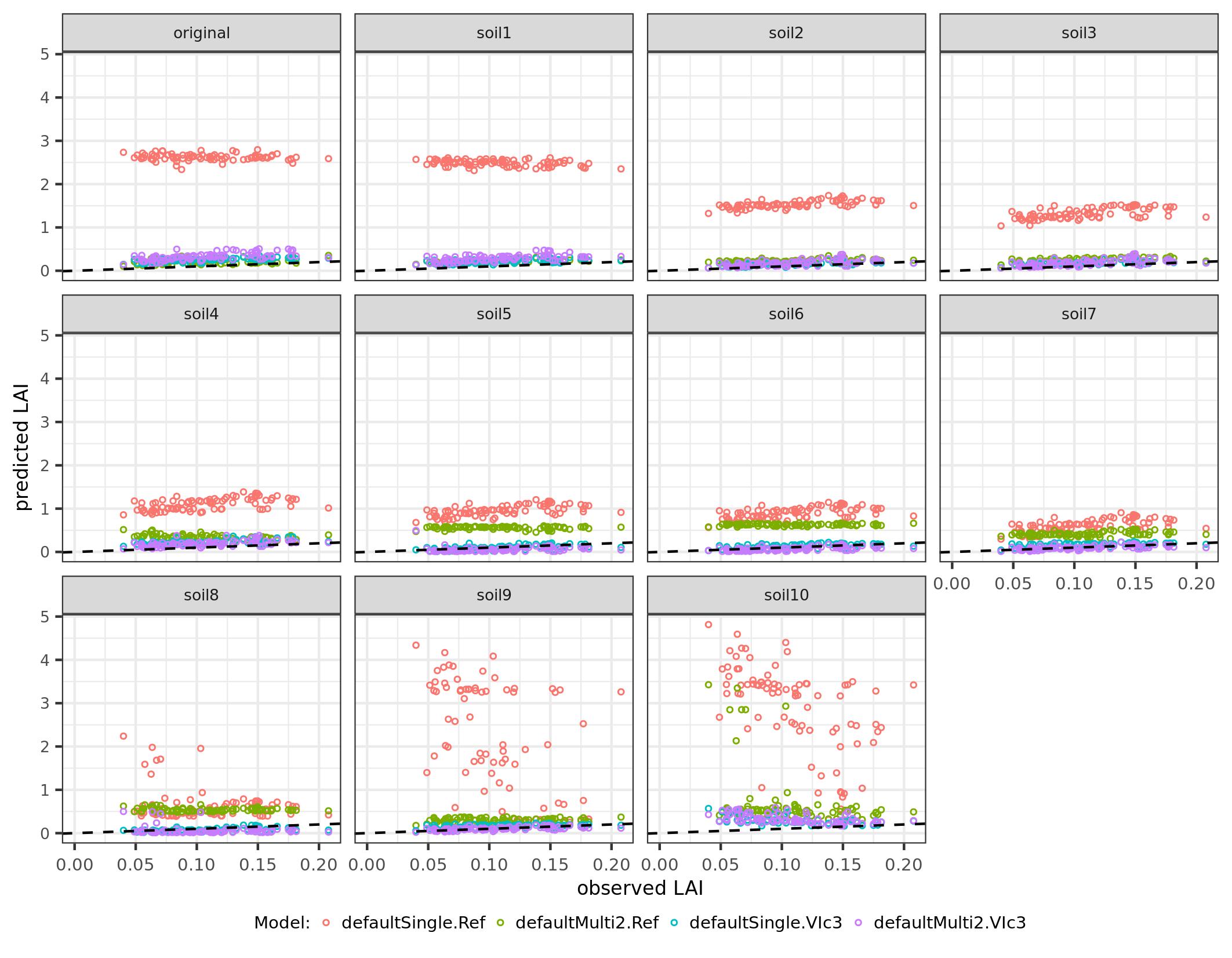


Figure S3 Observed LAI against predicted LAI for different soil backgrounds. The predicted LAI was retrieved using different RFR models (i.e., defaultSingle.Ref, defaultMulti2.Ref, defaultSingle.VIc3, defaultMulti2.VIc3) from (semi-)experimental multispectral data for different soil backgrounds at early growth stage (i.e., seedling growth stage, DAS=18 in Exp16). The “original” stands for the experimental multispectral images with original background, while “soil1” to “soil10” represent the semi-experimental multispectral images with test soil backgrounds.


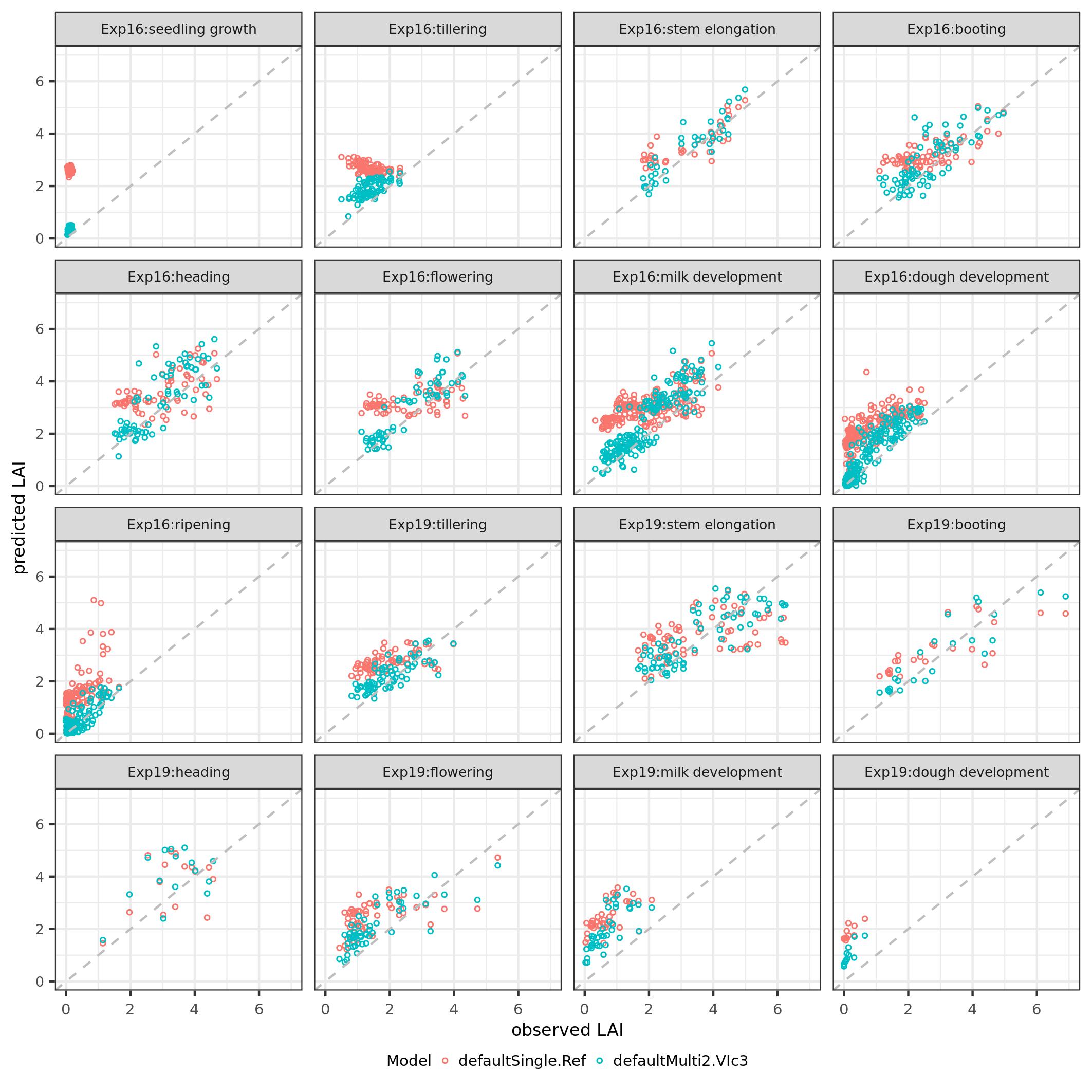


Figure S4 Observed LAI against predicted LAI at different growth stages. LAI was predicted with two RFR models (i.e., defaultSingle.Ref, defaultMulti2.VIc3) from real multispectral data at different growth stages (i.e., seedling growth, tillering, stem elongation, booting, heading, flowering, milk development, dough development, ripening) in two field experiments (i.e., Exp16, Exp19).


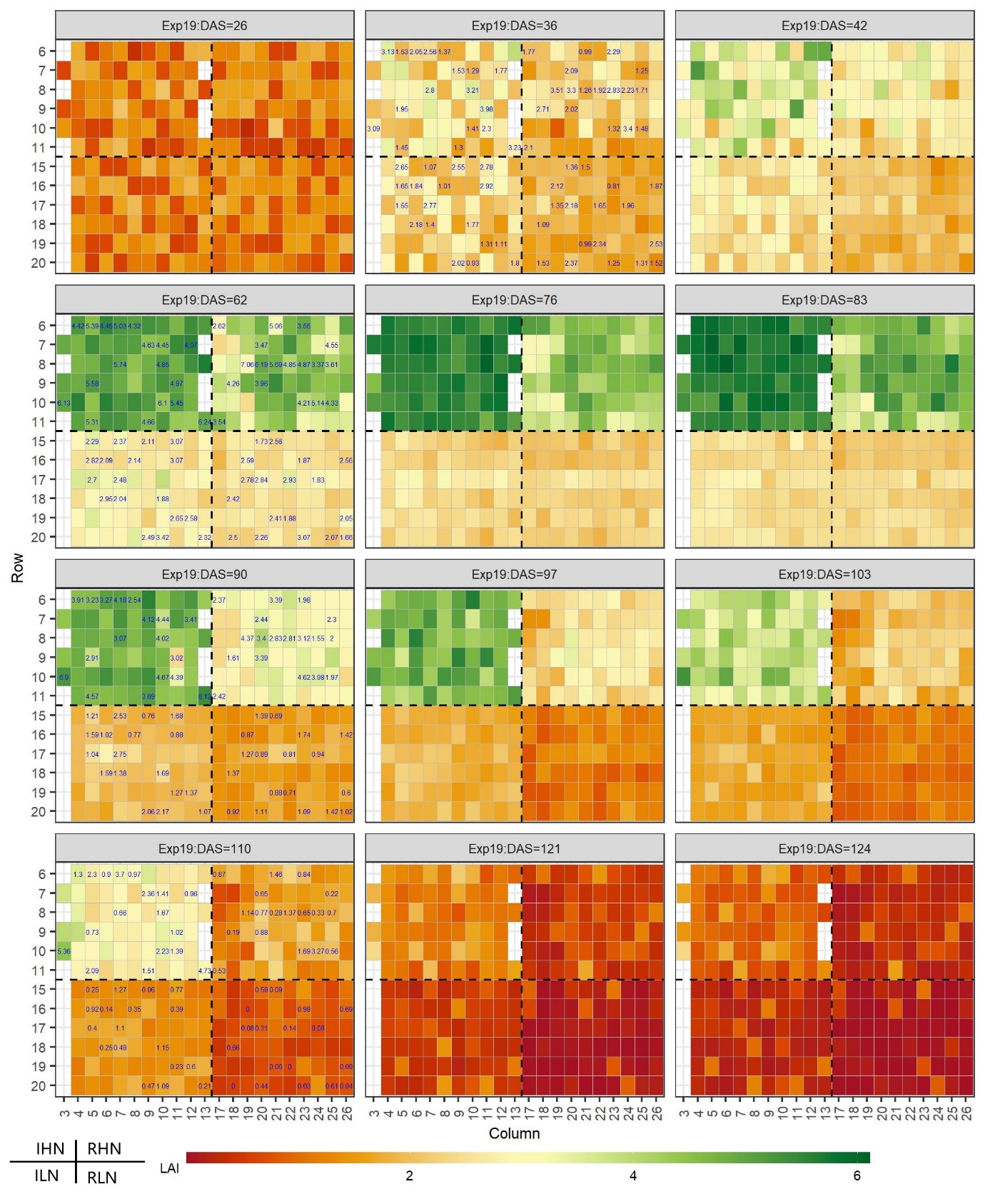


Figure S5 Predicted LAI for different phenotyping dates in Exp19. The predicted LAI was retrieved with the best RFR model (defaultMulti2.VIc3) using experimental multispectral data captured with the UAV platform. The plots with number of the top indicate those designed for UAV-based phenotyping, while the number denotes the observed LAI from corresponding plots used for destructive harvests. Only 72 plots at four dates have observed LAI as biophysical measurements were only conducted in these plots. Row and Column are used to locate the position of the plot in the field. The four blocks correspond to the four water-nitrogen treatments, i.e., irrigated high nitrogen (IHN), irrigated low nitrogen (ILN), rainfed high nitrogen (RHN), and rainfed low nitrogen (RLN).


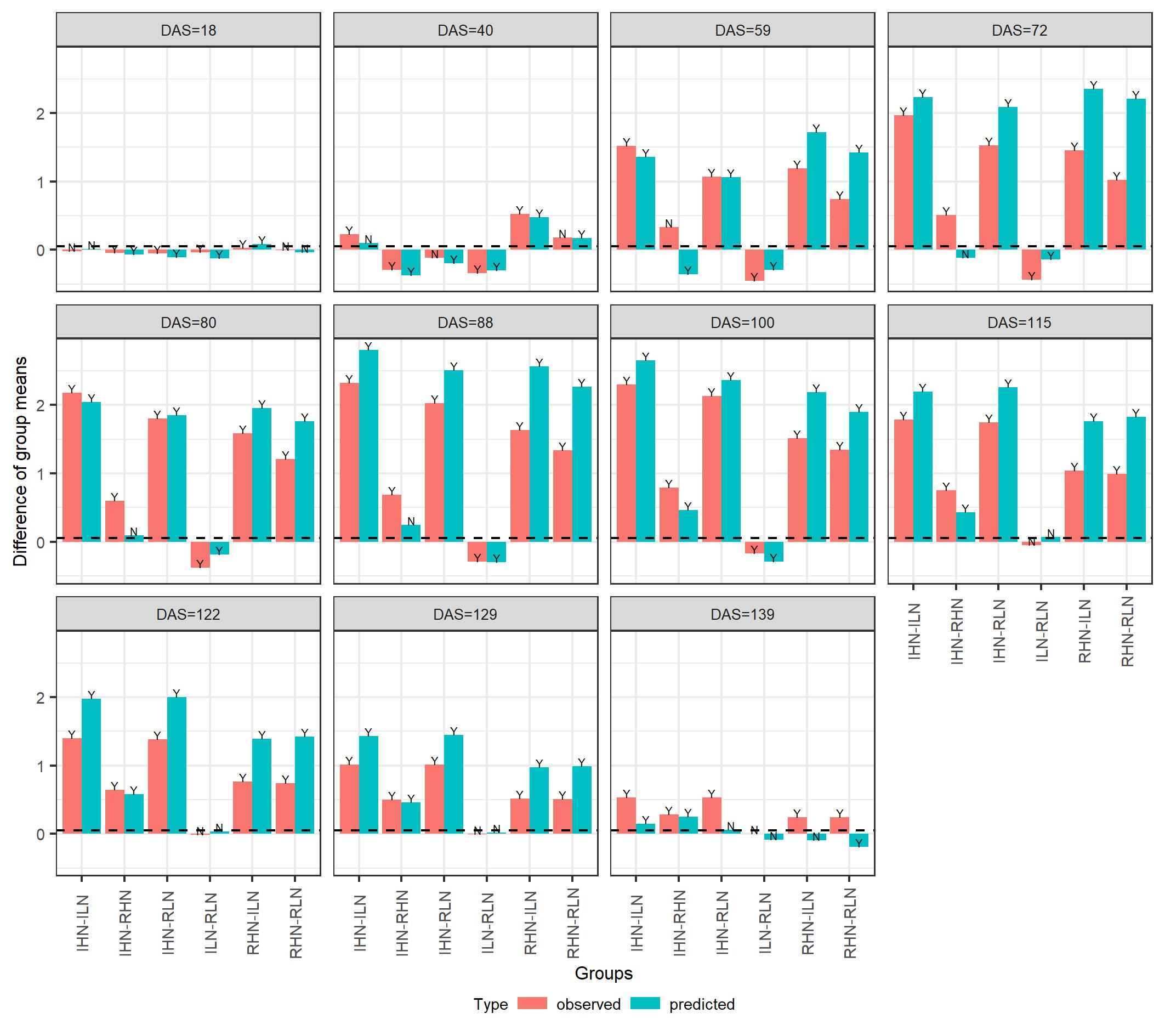


Figure S6 Difference for group means of any two water-nitrogen treatments in Exp16. The red bar indicates the difference of group means calculated from observed LAI. The blue bar indicates the difference of group means calculated from predicted LAI retrieved with the best RFR model (defaultMulti2.VIc3) using experimental multispectral data captured with the UAV platform. The text above the bar indicates if the group means are significantly different at 95% confidence interval: “N” for insignificant, “Y” for significant. The red bar indicates the difference of group means calculated from observed LAI. The significance was calculated with the two-sample t-test in R3.6.0. The four water-nitrogen treatments include irrigated high nitrogen (IHN), irrigated low nitrogen (ILN), rainfed high nitrogen (RHN), and rainfed low nitrogen (RLN). There are 21 data points for each water-nitrogen treatment for each UAV-based phenotyping date.


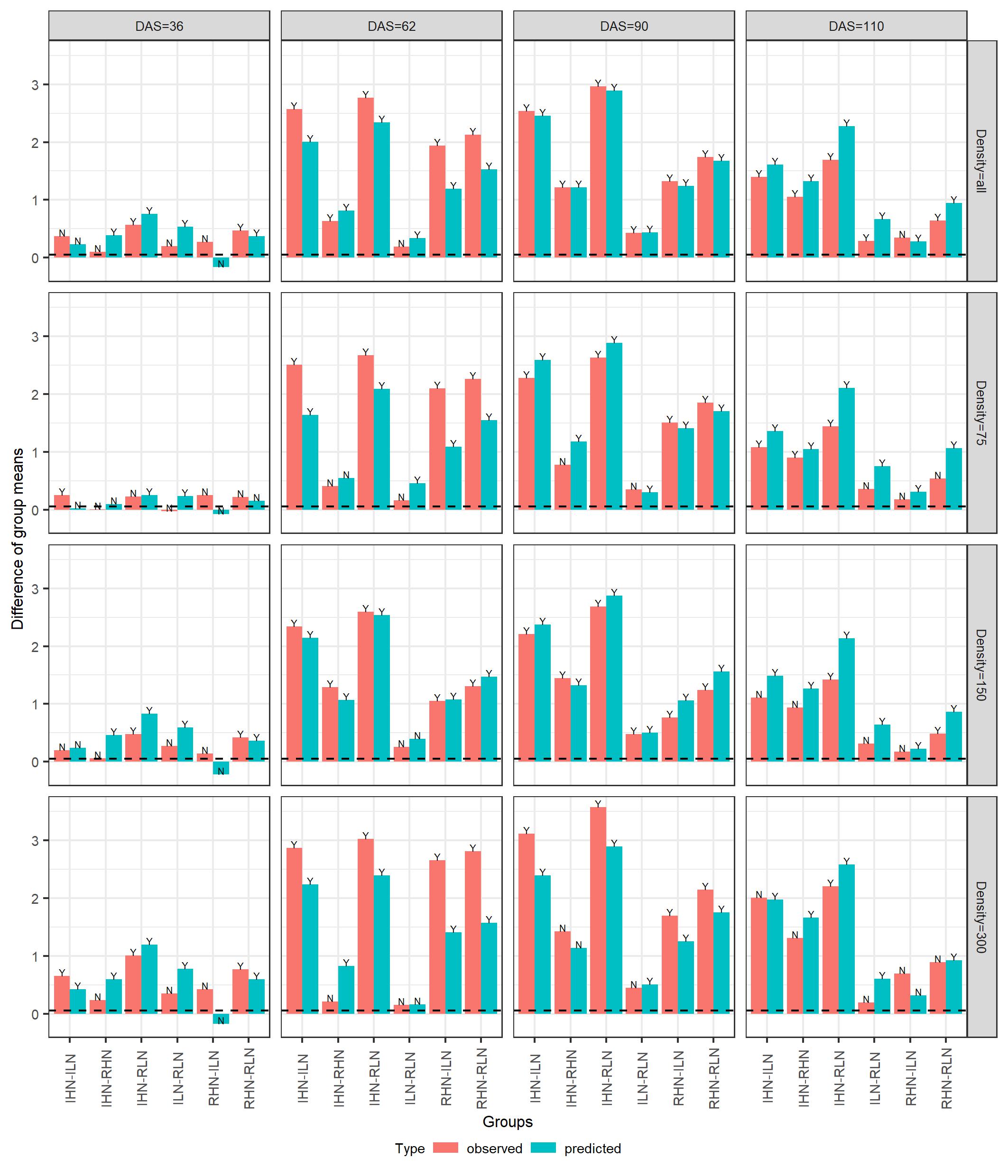


Figure S7 Difference for group means of any two water-nitrogen treatments in Exp19. The blue bar indicates the difference of group means calculated from predicted LAI retrieved with the best RFR model (defaultMulti2.VIc3) using experimental multispectral data captured with the UAV platform. The text above the bar indicates if the group means are significantly different at 95% confidence interval: “N” for insignificant, “Y” for significant. The red bar indicates the difference of group means calculated from observed LAI. The significance was calculated with the two-sample t-test in R3.6.0. The four water-nitrogen treatments include irrigated high nitrogen (IHN), irrigated low nitrogen (ILN), rainfed high nitrogen (RHN), and rainfed low nitrogen (RLN). For each water-nitrogen treatment at each UAV-based phenotyping date, there are 18 data points and six for each planting density treatment.


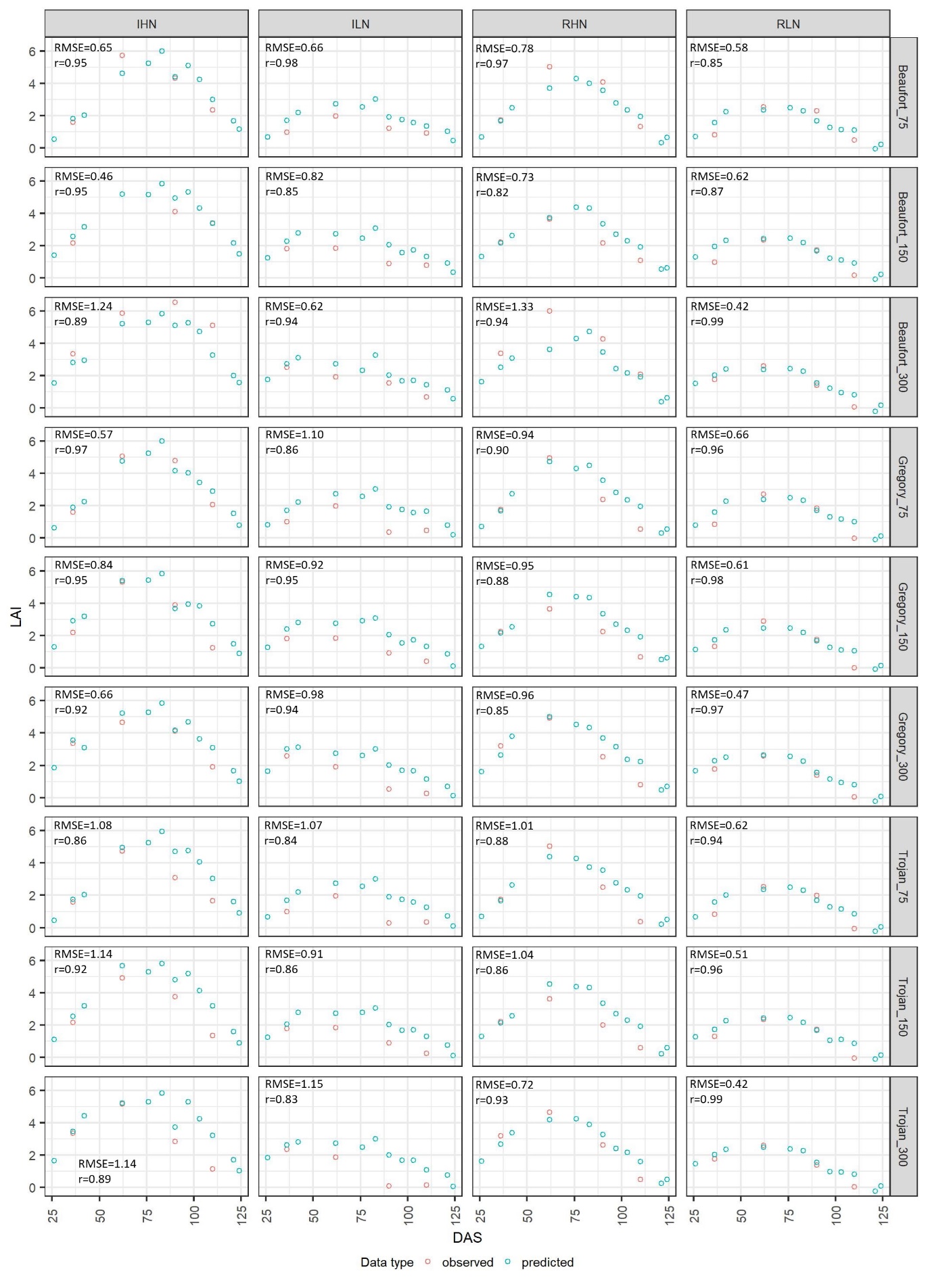


Figure S8 The dynamics of genotype-specific observed LAI and predicted LAI over growing season for Exp19. The red symbols indicate the genotype-specific values of observed LAI obtained from destructive harvest. The blue symbols correspond to the genotype-specific values of predicted LAI retrieved with the best RFR model (defaultMulti2.VIc3) using experimental multispectral data captured with the UAV platform. The genotype-specific values of LAI were calculated with the Best Linear Unbiased Prediction (BLUP) model. The four water-nitrogen treatments include irrigated high nitrogen (IHN), irrigated low nitrogen (ILN), rainfed high nitrogen (RHN), and rainfed low nitrogen (RLN).


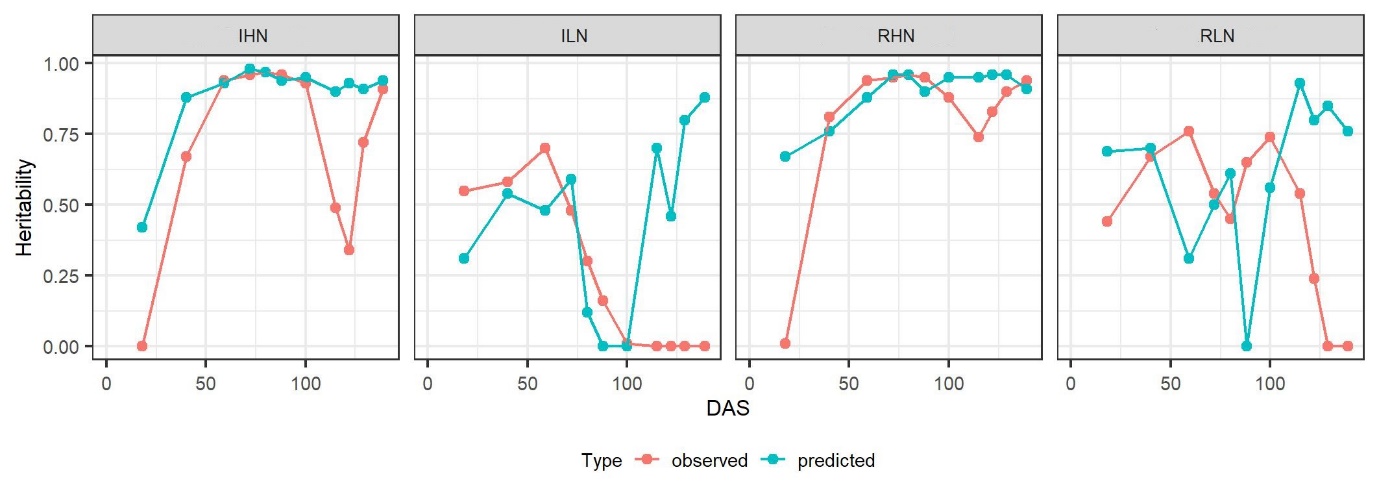


Figure S9 The changing heritability of observed LAI and predicted LAI under different water-nitrogen treatments for Exp16. DAS denotes days after sowing. The four water-nitrogen treatments include: irrigated high nitrogen (IHN), irrigated low nitrogen (ILN), rainfed high nitrogen (RHN), and rainfed low nitrogen (RLN). The predicted LAI were retrieved with the best RFR model (defaultMulti2.VIc3) using experimental multispectral data captured with the UAV platform. The heritability was calculated with the Best Linear Unbiased Prediction (BLUP) model.


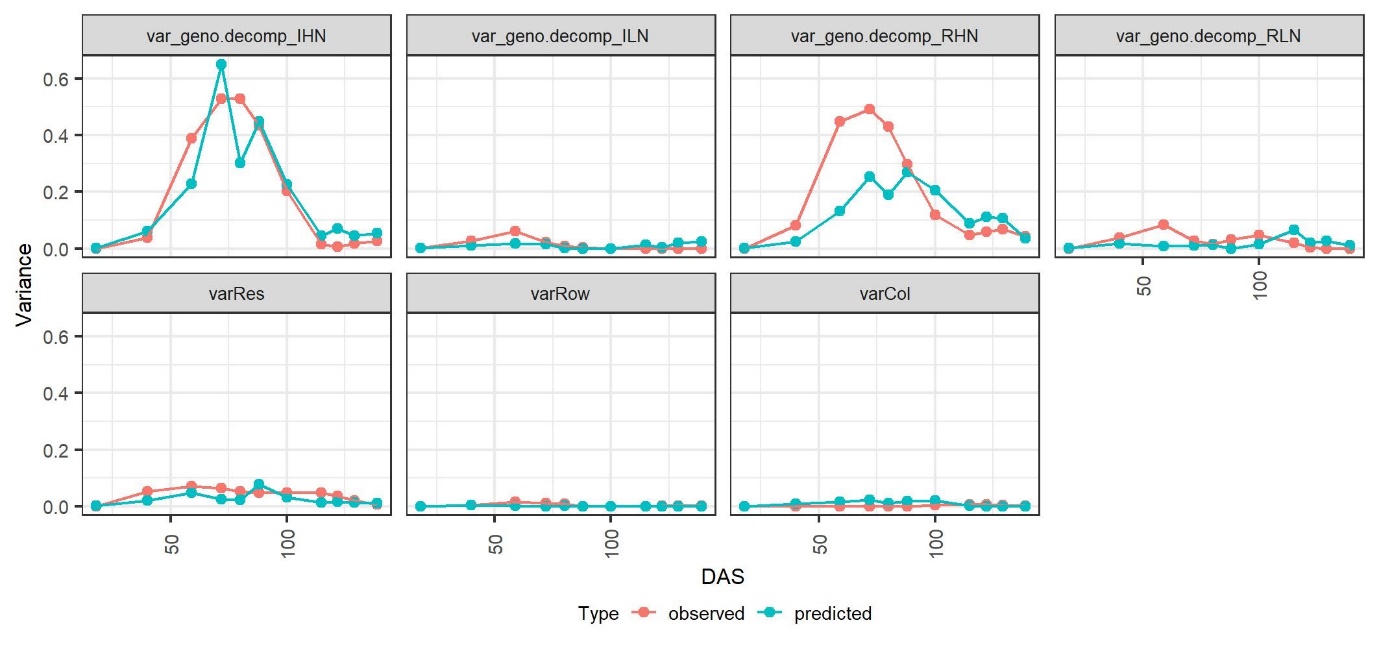


Figure S10 The changing variance of each component of observed LAI and predicted LAI for Exp16. DAS denotes days after sowing. The predicted LAI were retrieved with the best RFR model (defaultMulti2.VIc3) using experimental multispectral data captured with the UAV platform. The variance components were calculated with the Best Linear Unbiased Prediction (BLUP) model.


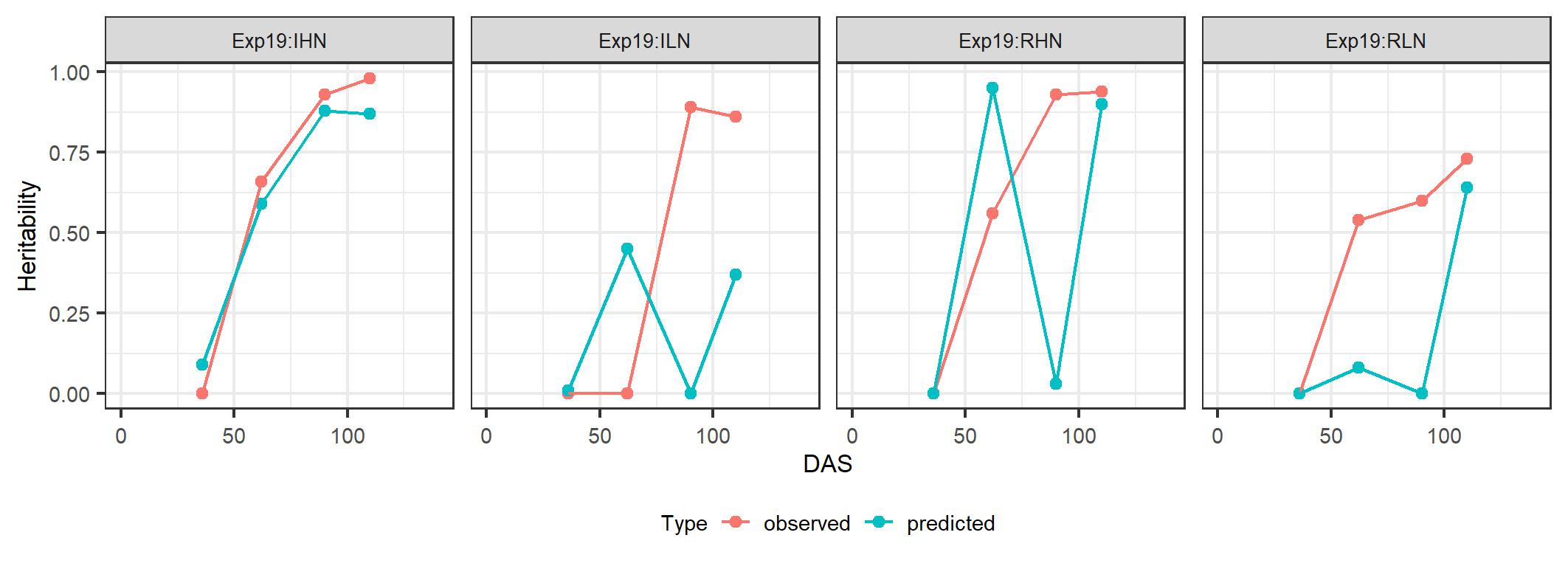


Figure S11 The changing heritability of observed LAI and predicted LAI under different water-nitrogen treatments for Exp19. DAS denotes days after sowing. The four water-nitrogen treatments include: irrigated high nitrogen (IHN), irrigated low nitrogen (ILN), rainfed high nitrogen (RHN), and rainfed low nitrogen (RLN). The predicted LAI were retrieved with the best RFR model (defaultMulti2.VIc3) using experimental multispectral data captured with the UAV platform. The heritability was calculated with the Best Linear Unbiased Prediction (BLUP) model.


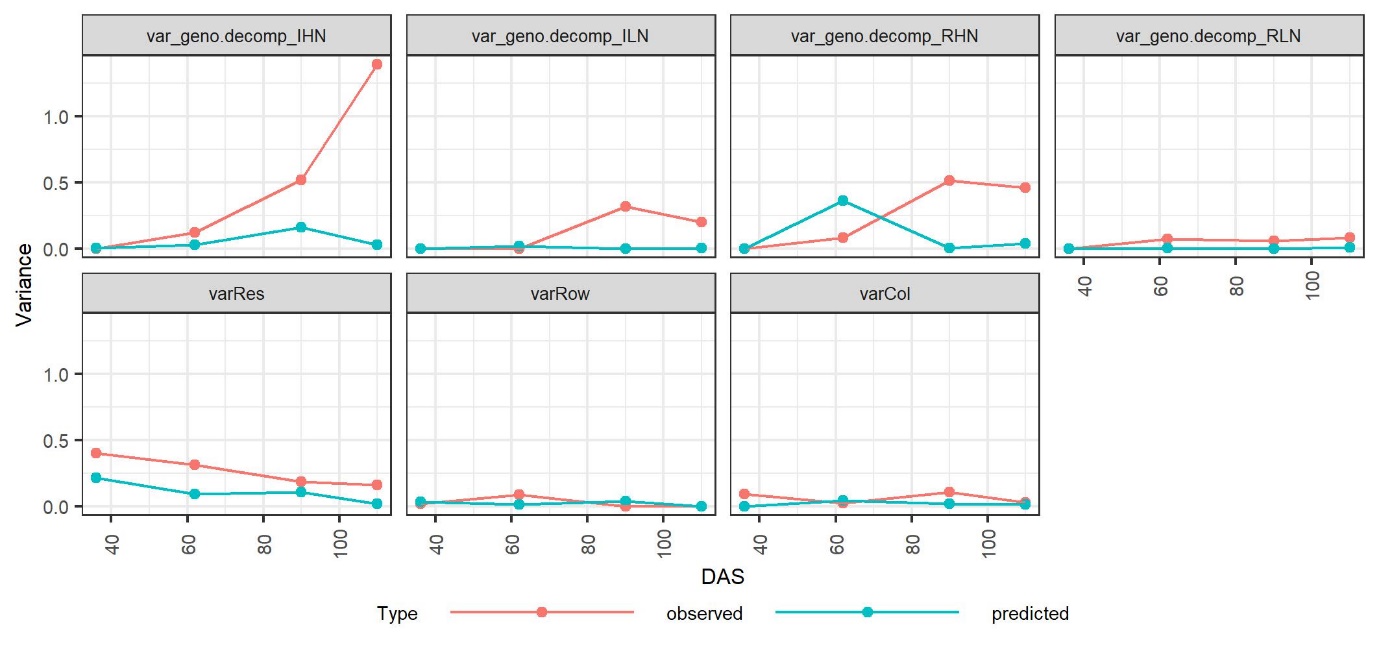


Figure S12 The changing variance of each component of observed LAI and predicted LAI for Exp19. DAS denotes days after sowing. The predicted LAI were retrieved with the best RFR model (defaultMulti2.VIc3) using experimental multispectral data captured with the UAV platform. The variance components were calculated with the Best Linear Unbiased Prediction (BLUP) model.


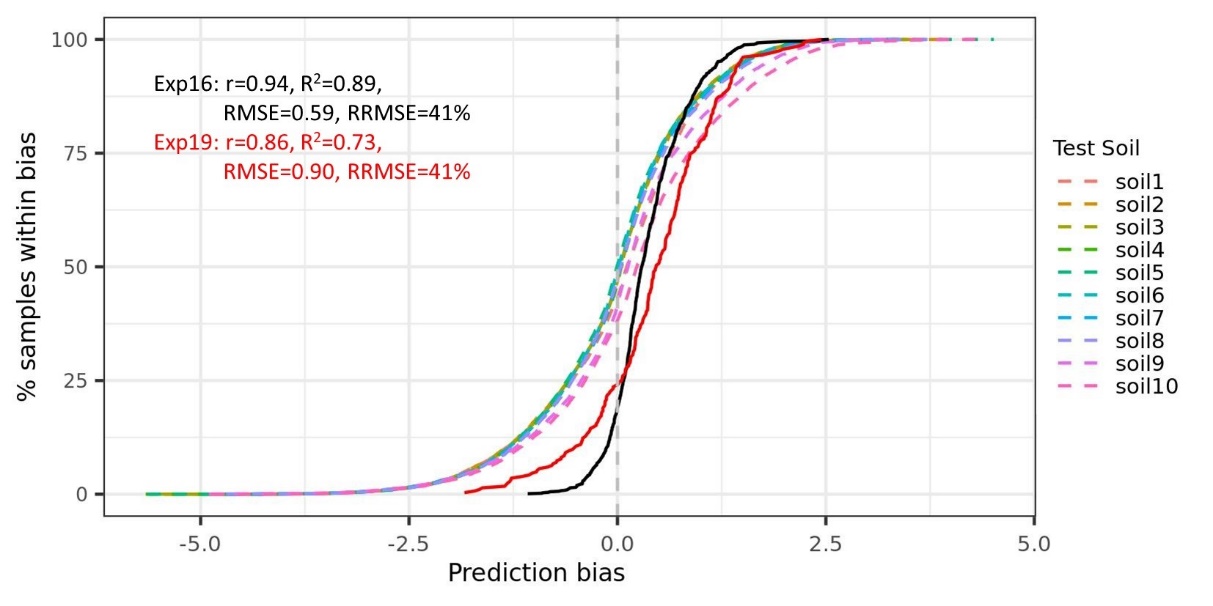


Figure S13 Empirical cumulative density distribution of LAI prediction bias. The bias indicates the difference of observed LAI subtracted from LAI retrieved with the defaultMulti2.VIc3 model. The dashed lines represent the theoretical performance on the synthetic test sets for different soil backgrounds (i.e., soil1 to soil10). The black and red solid lines represent the practical performance on real experimental data in Exp16 and Exp19, respectively.

# Supplementary tables

Table S1 Soil attributes of selected soil samples. The Sample_ID is the unique sample identification that can be used to search the detailed information of these soil samples excluded here in the Biomes of Australian Soil Environment (BASE) database (<https://data.bioplatforms.com/organization/australian-microbiome>). In the locations, the abbreviation “NP” stands for National Park.

| Soil background | Sample_ID | Collected  date | Latitude | Longitude | Location | Sand (%) | Silt (%) | Clay (%) | Color |
| --- | --- | --- | --- | --- | --- | --- | --- | --- | --- |
| soil1 | 102.100.100/13279 | 2013-10-25 | -39.65 | 143.97 | King Island | 48.04 | 7.48 | 44.49 | black |
| soil2 | 102.100.100/12526 | 2014-05-01 | -30.20 | 149.59 | Narrabri | 25.39 | 28.49 | 46.12 | grey brown |
| soil3 | 102.100.100/8141 | 2014-05-01 | -30.20 | 149.60 | Narrabri | 30.62 | 22.13 | 47.25 | grey |
| soil4 | 102.100.100/9464 | 2013-07-09 | -35.59 | 148.91 | Namadgi NP | 66.98 | 18.63 | 14.39 | brown |
| soil5 | 102.100.100/8515 | 2013-04-22 | -12.82 | 132.80 | Kakadu NP | 84.76 | 4.08 | 11.16 | light brown |
| soil6 | 102.100.100/8079 | 2013-08-06 | -30.17 | 115.21 | Mt Lesueur NP | 34.37 | 14.62 | 51.11 | brown |
| soil7 | 102.100.100/12578 | 2014-02-17 | -35.88 | 149.01 | Namadgi NP | 58.24 | 28.56 | 13.21 | grey brown |
| soil8 | 102.100.100/9431 | 2013-05-09 | -35.60 | 148.95 | Namadgi NP | 74.19 | 11.85 | 13.95 | grey yellow |
| soil9 | 102.100.100/8135 | 2013-12-08 | -33.88 | 119.92 | Fitzgerald River NP | 94.05 | 2.00 | 3.95 | grey |
| soil10 | 102.100.100/12436 | 2013-07-17 | -42.06 | 148.30 | Freycinet NP | 98.10 | 0.00 | 1.90 | white |
